# Systems pharmacology reveals the unique mechanism features of Shenzhu Capsule for treatment of ulcerative colitis in comparison with synthetic drugs

**DOI:** 10.1038/s41598-018-34509-1

**Published:** 2018-11-01

**Authors:** Wuwen Feng, Hui Ao, Shijun Yue, Cheng Peng

**Affiliations:** 1 School of Pharmacy, Chengdu University of Traditional Chinese Medicine, Chengdu, China; 2 College of Pharmacy and Shaanxi Collaborative Innovation Center of Chinese Medicinal Resources Industrialization, Shaanxi University of Chinese Medicine, Xianyang, China; 3 State Key Laboratory Breeding Base of Systematic Research, Development and Utilization of Chinese Medicine Resources, Chengdu University of Traditional Chinese Medicine, Chengdu, China

## Abstract

In clinic, both synthetic drugs and Shenzhu Capsule (SZC), one kind of traditional Chinese medicines (TCMs), are used to treat ulcerative colitis (UC). In our study, a systems pharmacology approach was employed to elucidate the chemical and mechanism differences between SZC and synthetic drugs in treating UC. First, the compound databases were constructed for SZC and synthetic drugs. Then, the targets of SZC were predicted with on-line tools and validated using molecular docking method. Finally, chemical space, targets, and pathways of SZC and synthetic drugs were compared. Results showed that atractylenolide I, atractylone, kaempferol, etc., were bioactive compounds of SZC. Comparison of SZC and synthetic drugs showed that (1) in chemical space, the area of SZC encompasses the area of synthetic drugs; (2) SZC can act on more targets and pathways than synthetic drugs; (3) SZC can not only regulate immune and inflammatory reactions but also act on ulcerative colitis complications (bloody diarrhea) and prevent UC to develop into colorectal cancer whereas synthetic drugs mainly regulate immune and inflammatory reactions. Our study could help us to understand the compound and mechanism differences between TCM and synthetic drugs.

## Introduction

Ulcerative colitis (UC) is a group of chronic inflammatory disorders of gastrointestinal tract characterized by intestinal inflammation and mucosal damage^1^. Together with Crohn’s disease and other diseases, they are classified under the category of inflammatory bowel disease (IBD). Epidemiologic studies show that the prevalence of UC is about 100–200 per 100,000 in Western countries^2^. In clinic, UC is associated with recurrent attacks that may last several months and even to years, and it can impact on patients’ overall ability and everyday life^3^. In addition, UC is also associated with a predisposition to develop to more severe colorectal cancer^4^. In China, both traditional Chinese medicines (TCMs) and synthetic drugs such as glucocorticoids and sulfasalazine have been widely used to treat UC^2,5^.

In TCM, UC can be categorized as *jiuli* (enduring dysentery), *changbi* (intestinal impediment), *daxiaxie* (great conglomeration diarrhea), *xiexie* (diarrhea), *tongxie* (painful diarrhea), *chibaili* (red and white dysentery), and *bianxue* (hemafecia)^6^. In clinic, many TCM formulas have been proven to be effective in treating UC^6–9^. Of these medicines, ShenZhu Capsule (SZC) has shown great potential in treating UC (*xiexie* syndrome) and other gastrointestinal diseases^10^. This formula contains two herbal medicines, Renshen (*Ginseng radix et rhizoma*, the dried roots of *Panax ginseng* C.A. Mey.) and Baizhu (*Atractylodis macrocephalae rhizoma*, the dried roots of *Atractylodes macrocephala* Koidez.), both of which have been demonstrated to exhibit good effects in treating UC^11,12^. Although the therapeutic effects of SZC has been confirmed, the active compounds in SZC and specific molecular mechanisms of SZC in treating UC remain unclear. Therefore, it is of great significance to identify the bioactive compounds and mechanisms of SZC.

**Figure 1:**
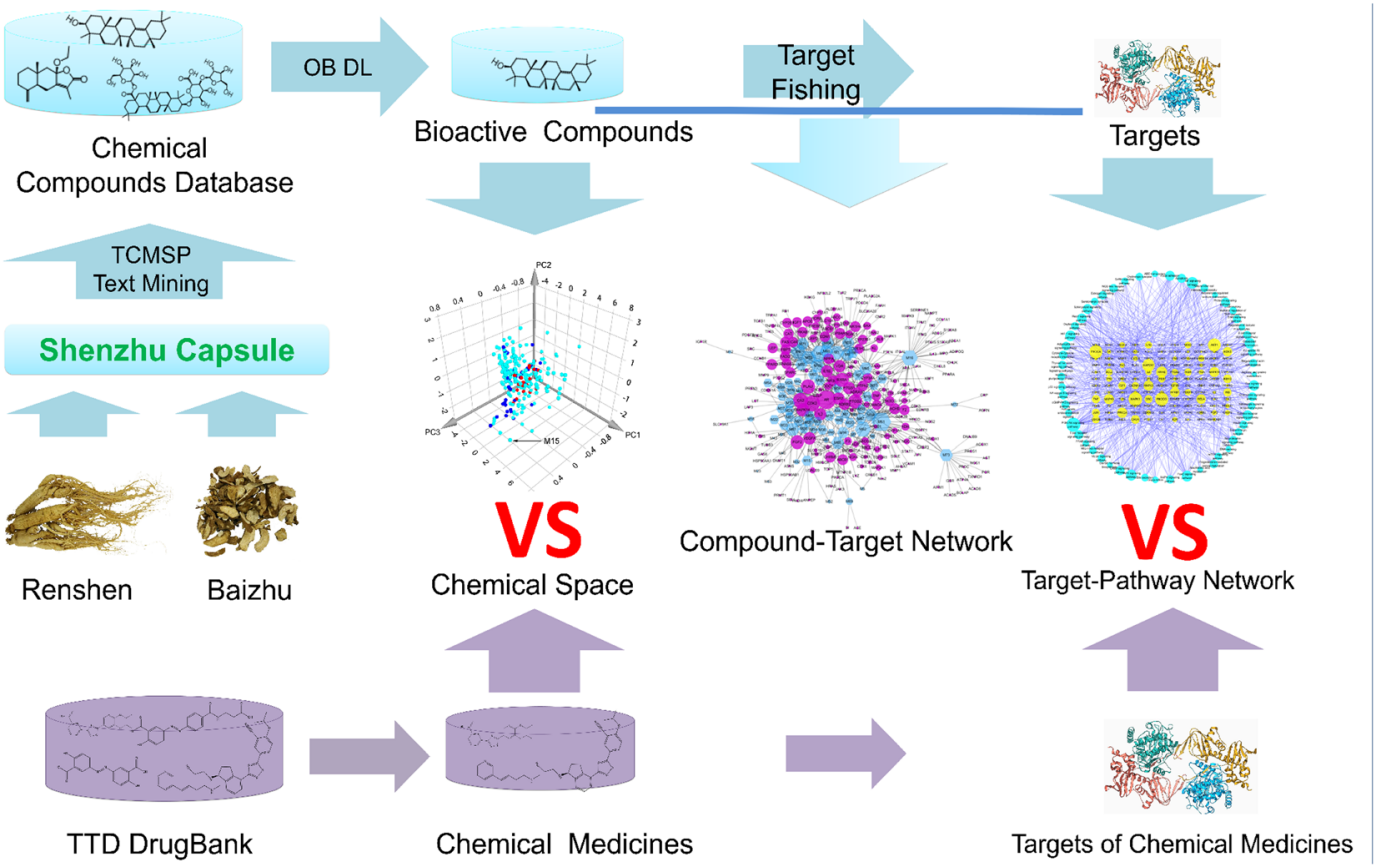
Schematic diagram of the systems pharmacology-based strategy for unraveling the bioactive compounds and mechanism features of Shenzhu Capsule (SZC).

System pharmacology is an efficient tool to study the active compounds and mechanisms of complex TCM. With the introduction of system pharmacology, it enables us to discover bioactive ingredients and drug targets, to reveal the mechanisms of action, and to explore the scientific evidences of numerous herbs and herbal formulae in TCM on the basis of complex biological system of human body^13,14^. For this reason, it has been used successfully in study the mechanisms of TCM, such as Xipayi KuiJie and Si-Jun-Zi-Tang^15,16^. In our present work, a standard system pharmacology approach was adopted to determine the active compounds and mechanisms of SZC in treating UC^17^. First, we obtained the compounds of SZC by data retrieving from on-line databases and manual supplement with text-mining method. Then, we used two parameters including OB and DL to screen active compounds. And then, we applied tools such as BATMAN-TCM to predict the targets of SZC and molecular docking was used to validate the targets. Finally, Compound-Target network and Target-Pathway network was constructed to visualize and analysis the mechanisms of SZC. In addition, to obtain the mechanism features of SZC in treating UC, we also compared the chemical space, targets, and pathways differences between SZC and synthetic drugs by principal component analysis (PCA) and KEGG (Kyoto Encyclopedia of Genes and Genomes) pathway analysis. The flowchart of our study is shown in Fig. 1.

## Results

### Chemical compounds of SZC and synthetic drugs

By retrieving of chemical compounds of Renshen and Baizhu from data bases and manual supplement from literatures, a total of 322 compounds in SZC were obtained, including 261 compounds that only exist in Renshen, 46 compounds that only exist in Baizhu, and 5 compounds that exist in both herbal medicines (Supplementary Table S1). The major components of Renshen are ginsenosides and the main components of Baizhu are lactones. In addition, 22 small molecular drugs that are already used in clinic or in clinical trial were retrieved from DrugBank (Supplementary Table S2). Then, OB and DL, two ADME related parameters were applied to screen bioactive compounds in SZC. In addition, compounds that with poor OB and DL yet have been proved to be bioactive were also preserved in our study. As a result, 73 bioactive compounds in SZC were obtained; of which 9 exist only in Baizhu, 63 exist only in Renshen and 1 exists in both Renshen and Baizhu (Supplementary Table S3).

### Active compounds in SZC

In Baizhu, only 5 compounds satisfied the criteria of OB ≥30% and DL ≥0.18, these compounds were (24 *S*)-24-propylcholesta-5-ene-3*β*-ol (OB = 36.23, DL = 0.78), 3*β*-acetoxyatractylone (OB = 54.07, DL = 0.22), 8*β*-ethoxy atractylenolide III (OB = 35.95, DL = 0.21), atractylenolide III (OB = 68.11, DL = 0.17) and *α*-amyrin (OB = 39.51, DL = 0.76). Most of them have been proved to exhibit potent pharmacological activities. For example, *α*-amyrin has been proved to exhibit the ability to attenuate dextran sulfate sodium-induced colitis^18^, atractylenolide III showed anti-inflammatory activity and anti-gastric ulcer activity^19,20^. Other 5 compounds in Baizhu that did not meet the criteria were also preserved, including atractylenolide I (OB = 37.37, DL = 0.15), atractylenolide II (OB = 47.5, DL = 0.15), atractylone (OB = 41.1, DL = 0.13), *β*-caryophyllene (OB = 29.7, DL = 0.09) and *β*-eudesmol (OB = 26.09, DL = 0.10). Those compounds were included because of their bioactivities. For example, *β*-caryophyllene could attenuate dextran sulfate sodium-induced colitis^21^, atractylone could attenuate allergic inflammation^22^.

In Renshen, 17 compounds that satisfied screening parameters were extracted. Some of those compounds have been proved to exhibit potent pharmacological effects. For example, stigmasterol (OB = 43.83, DL = 0.76) and kaempferol (OB = 41.88, DL = 0.24) can suppress inflammation of dextran sulfate sodium-induced colitis^23,24^, *β*-sitosterol (OB = 36.91, DL = 0.75) could inhibit TNBS-induced colitis^25^. It should be noted that Renshen contains a large amount of ginsenosides, and most of them did not meet the criteria of OB ≥30% and DL ≥0.18. However, most of them have been reported to exhibit potent pharmacological effects. For example, ginsenoside Rb1 (OB = 6.24, DL = 0.04) could attenuate TNF-*α*-induced and free fatty acids-induced inflammatory injury^26^, ginsenoside Rg1 (OB = 11.21, DL = 0.23) could attenuate the inflammatory response in DSS-induced mice colitis^27^, ginsenoside Rc (OB = 8.13, DL = 0.04) could attenuate inflammatory symptoms of gastritis, hepatitis and arthritis^28^. In addition, ginsenoside Rg1 and ginsenoside Rb1 have been chosen as the quality control markers for Renshen in Chinese Pharmacopoeia^29^. Thus, those compounds were selected for further analysis.

### Chemical space comparison of SZC and synthetic drugs

**Figure 2:**
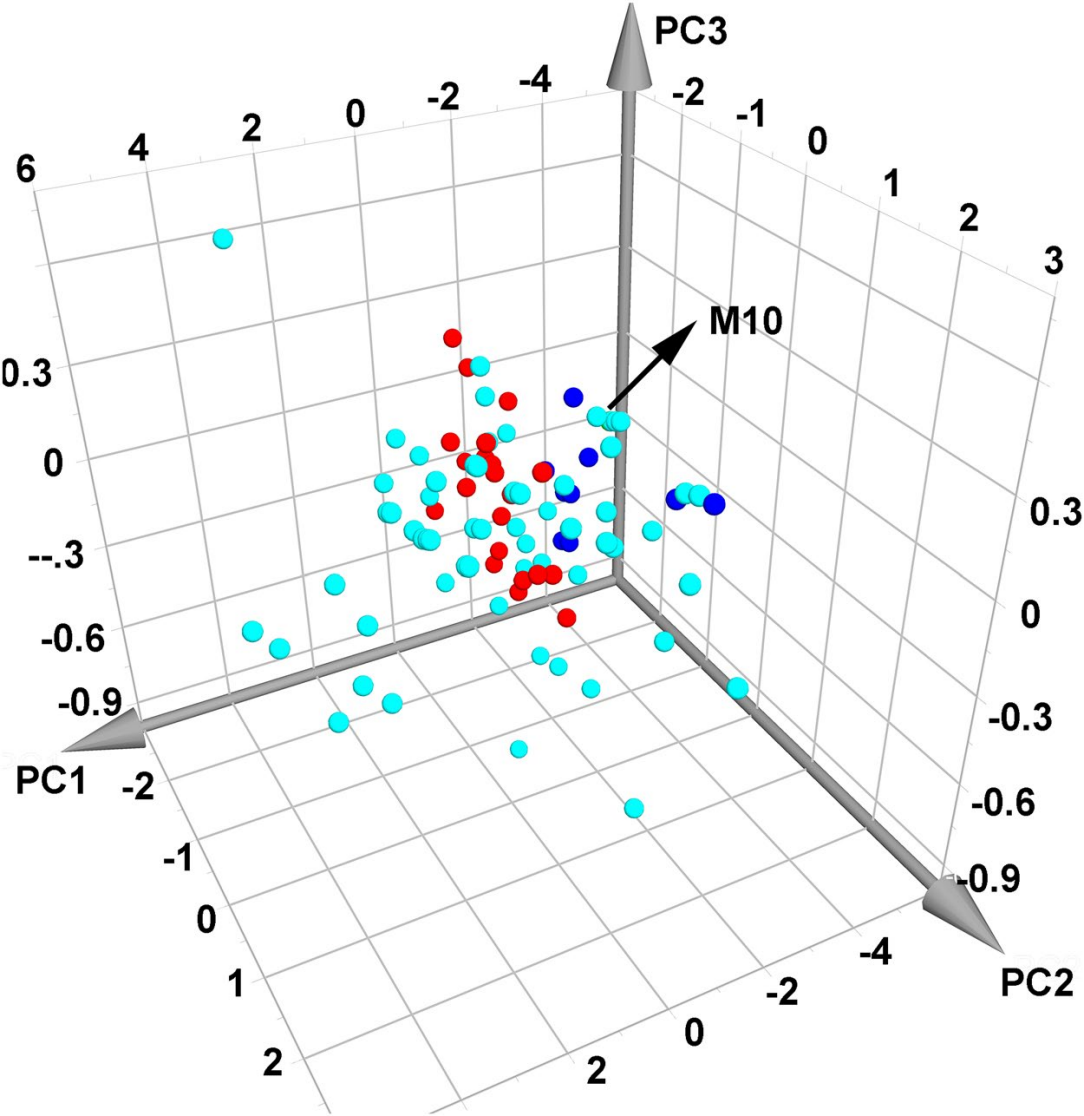
Comparison of the chemical space between ShenZhu Capsule and synthetic drugs. The cyan and blue balls represent compounds in Renshen and Baizhu, respectively. The red balls stand for synthetic drugs. The green ball (M10, nearly over shadowed by cyan balls) delineate common compounds in Renshen and Baizhu.

To investigate the molecular diversity of compounds in SZC and to visualize the chemical difference between SZC compounds and synthetic drugs, PCA was performed using 4 parameters including MW, Clogp, nHDon and nHAcc^14^. Results showed that PC1, PC2, PC3 account for 77.6%, 19.9%, 1.5% of total component, respectively. The compounds in SZC distribute broadly in chemical space (Fig. 2). Noteworthy, there are large overlaps between compounds in SZC and synthetic drugs, indicating that those compounds share similar chemical properties and might have similar biological properties. Still, there are many compounds in SZC that are distant from synthetic drugs in chemical space. For example, in PC1, synthetic drugs distributed in the ranges from about -3 to 0, while compounds from Baizhu distributed in the ranges from about -6 to -3. Compared with synthetic drugs, those compounds in Baizhu cover different chemical space and exhibited different chemical properties. The different chemical properties might confer different biological properties to those compounds. Thus, it would be meaningful to figure out those similar and different biological properties between SZC and synthetic drugs.

### Targets of SZC and synthetic drugs

By target fishing, 10 bioactive compounds in Baizhu were found to be associated with 67 UC-related target proteins (Supplementary Table S4). For example, *α*-amyrin may have potential to act on 16 targets including IL1B (interleukin 1 beta), NFKB1 (nuclear factor kappa B subunit 1). Interestingly, *α*-amyrin has been reported to be an inhibitor of IL1B, which might contribute to the therapeutic effects in UC treatment^18^. Similarly, by target fishing, *β*-eudesmol may have potential to act on 20 targets including IL1B, NFKB1, IL2 (interleukin 2), etc. Meanwhile, studies have also proved that *β*-eudesmol could suppress activation of NFKB^30^. *β*-caryophyllene, which may have potential act on 22 targets including IL2, NFKB1, NPPA (natriuretic peptide A), etc., has been proved to be able to regulate the expression of inflammation-related gene IL-1*β* in colon tissue^21^. Those studies indirectly proved the reliability of our target proteins.

For Renshen, a total of 64 bioactive compounds were found to be associated with 202 UC-related target proteins. These targets were PTPN2 (protein tyrosine phosphatase, non-receptor type 2), FGF2 (fibroblast growth factor 2), etc. For example, kaempferol were found to have potential act on targets such as TNF (tumor necrosis factor), PPARG (peroxisome proliferator activated receptor gamma), and studies have also proved that kaempferol could interact with TNF-*α* to modulate systemic inflammation and oxidative stress^31^. A noteworthy fact is that some targets such as F2 (thrombin) are not directly connected with major syndrome of UC but with the complication of UC (bloody diarrhea). By target fishing, some compounds in Renshen were found to be able to inhibit thrombin, such as kaempferol, frutinone A, deoxygomisin A, riboflavin. For these compounds, kaempferol has been proved to be able to inhibit thrombin^32^.

As a result, 210 UC-related targets interacting with 73 active compounds in SZC were obtained, meanwhile, 54 targets of synthetic drugs were also gathered (Supplementary Table S4).

### Compound-target interaction validation by molecular docking

**Figure 3:**
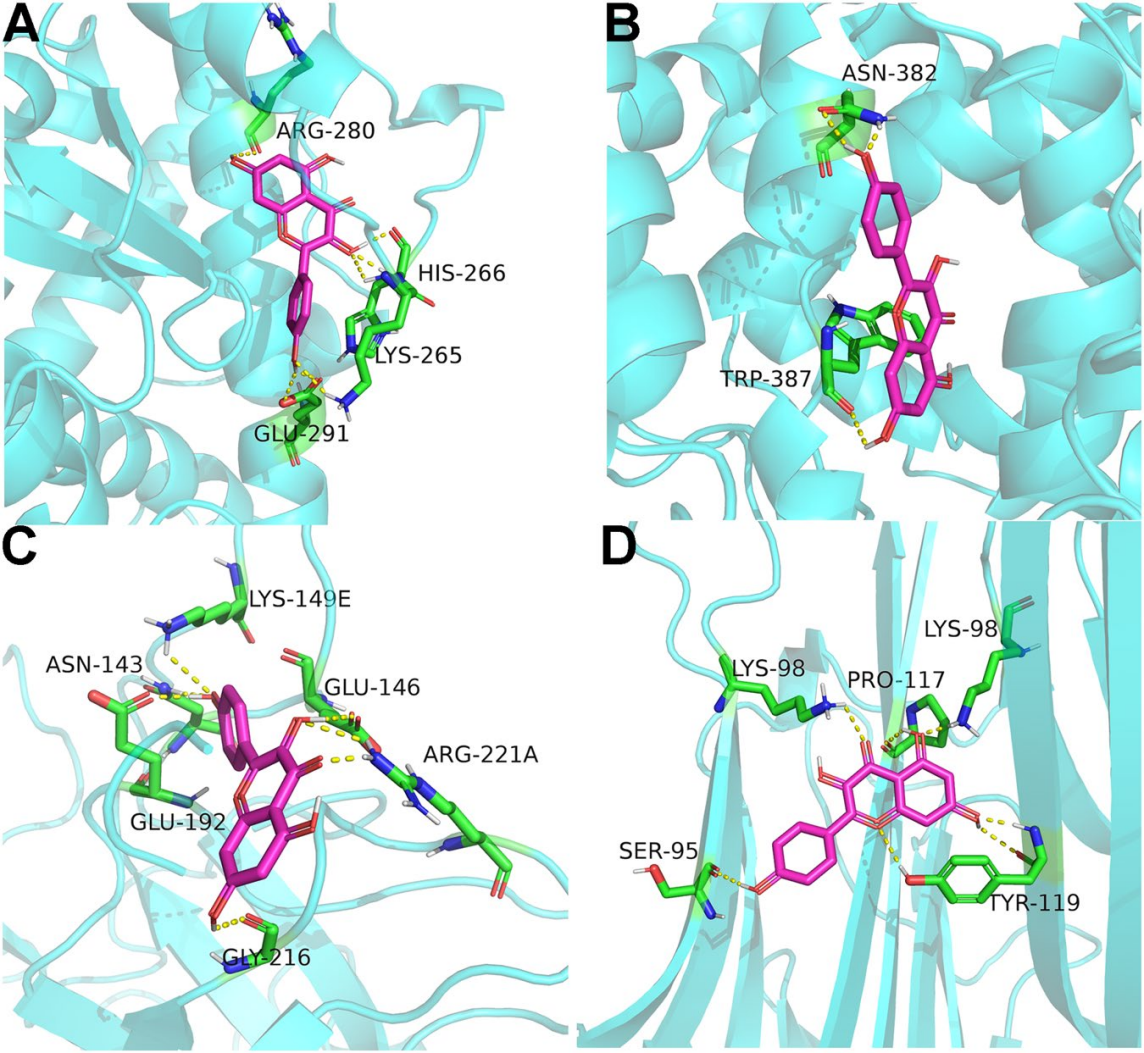
Docking modes of kaempferol and four target proteins. (**A**) peroxisome proliferator activated receptor gamma (PPARG), (**B**) prostaglandin-endoperoxide synthase 2 (PTGS2), (**C**) thrombin (F2), (**D**) tumor necrosis factor (TNF). Kaempferol and residues are shown in stick format, hydrogen bonds are shown as yellow dashed lines. Pink and green: carbon; red: oxygen; gray: hydrogen; blue: nitrogen.

To verify the reliability of targets, molecular docking was applied to view and calculate the compound-target binding interactions. The docking was calculated by docking of active compounds in SZC to known ligands binding site in predicted targets. Figure 3 shows the example of kaempferol binds to target proteins, including PPARG, prostaglandin-endoperoxide synthase 2 (PTGS2), F2, and TNF. Kaempferol can bind to amino acid residue ARG-280, HIS-206, LYS-265, GLU-291 in PPARG by hydrogen bonds (Fig. 3A). It can also bind to PTGS2 amino acid residue ASN-382 and TRP-387 by three hydrogen bonds (Fig. 3B). In general, docking scores with binding energy ≤−5.0 kcal/mol indicate good compound-target interaction^33,34^. The binding energy between kaempferol and PPARG, PTGS2, F2, TNF were −7.94, −7.80, −8.80, −6.84 kcal/mol, respectively, indicating that kaempferol could interact with PPARG, PTGS2, F2, and TNF.

### Targets comparison of SZC and synthetic drugs

**Figure 4:**
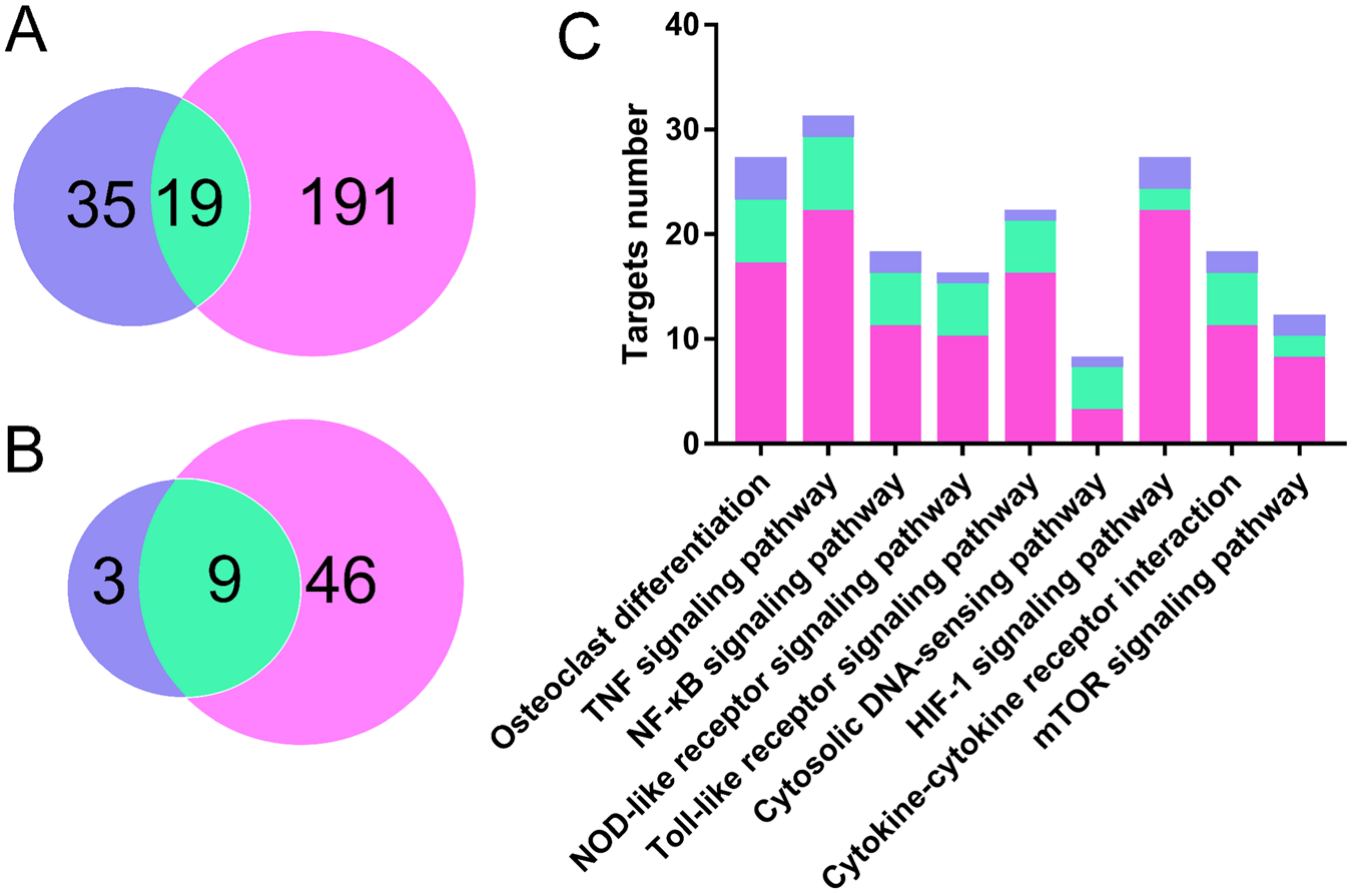
Comparison of the targets and pathways of synthetic drugs and Shenzhu Capsule (SZC). (**A**) Comparison of the number of targets for synthetic drugs and SZC (blue represents synthetic drugs, cyan delineates shared targets, light red stands for SZC). (**B**) Comparison of the number of pathways for synthetic drugs and SZC. (**C**) Number of targets for synthetic drugs and SZC in each pathway.

To compare the differences of the target proteins between SZC and synthetic drugs, we also retrieved the target proteins of synthetic drugs from TTD and Drugbank (Supplementary Table S5). Comparison of the targets showed that 191 targets belonged to SZC but not to synthetic drugs, and 35 targets belonged to synthetic drugs but not to SZC (Fig. 4A), including CXCL10 (C-X-C motif chemokine ligand 10), IL1A, RPS6KB1 (ribosomal protein S6 kinase B1), etc. Gene ontology (GO) and KEGG pathway analysis of the targets of synthetic drugs showed that they were highly related to immune response and primary immunodeficiency pathway, while the targets of SZC were related to broad functions and pathways (see below and Supplementary Figs 1 and 2). In addition, SZC and synthetic drugs share 19 targets, including TNF, IL1B, PPARG, etc. GO analysis and KEGG pathway analysis of these shared targets showed that they were highly related to inflammatory response with NF-*κ*B signaling pathway and TNF signaling pathway involved.

### The mechanism of SZC by Compound-Target network analysis

**Figure 5:**
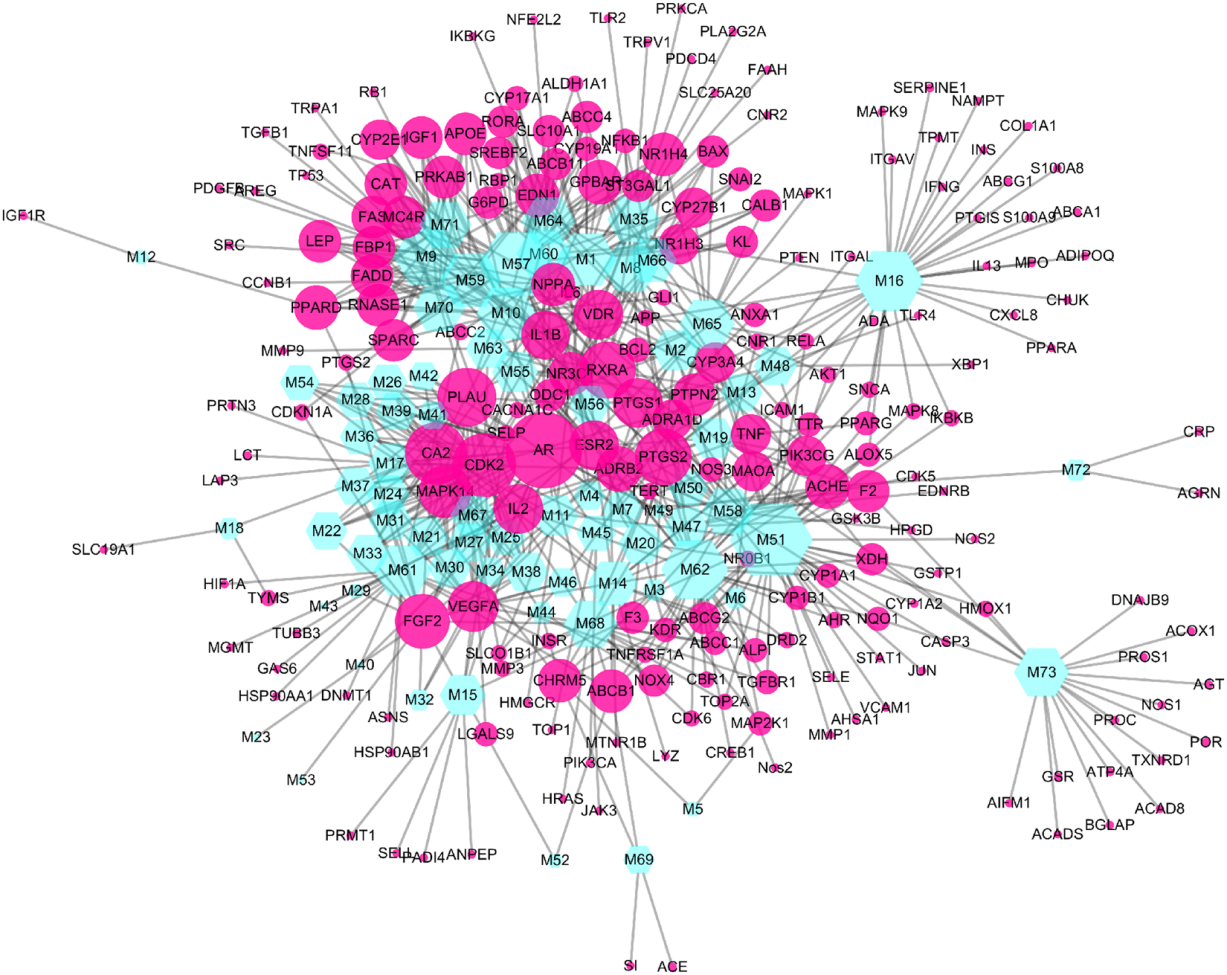
Compound-Target (C-T) network of Shenzhu Capsule. Nodes represent compounds and targets, and the node size is related to the degree of nodes.

To elucidate the therapeutic effects of SZC in treating UC at system level, two network analyses including Compound-Target (C-P) and Target-Pathway (T-P) analysis were performed. First, a C-T network is presented with the help of Cytoscape 3.5.1 (Fig. 5). The C-T network showed 740 interactions between 73 bioactive compounds and 210 targets. For the bioactive compounds, kaempferol (M51, degree = 46) exhibited the biggest number of interactions with the targets, followed by stigmasterol (M57, degree = 37) and dianthramine (M16, degree = 32). These compounds with high degrees demonstrated that single compound can target multiple receptors and were responsible for the high interconnectedness of C-T network. Noteworthy is that the compounds in SZC not only targeted on key modulators involved in UC (such as IL1B, TNF and TLR4), but also on other targets participated in UC complications such as bloody diarrhea (F2, thrombin). Another noteworthy thing is that most targets such as PLAU (plasminogen activator, urokinase), IL2, ABCB1 (ATP binding cassette subfamily B member 11), and PTGS2 were synergistically regulated by different compounds while some compounds could regulate different targets. These results demonstrated the multitargeting nature of SZC.

### The mechanism of SZC by Target-Pathway network analysis

**Figure 6:**
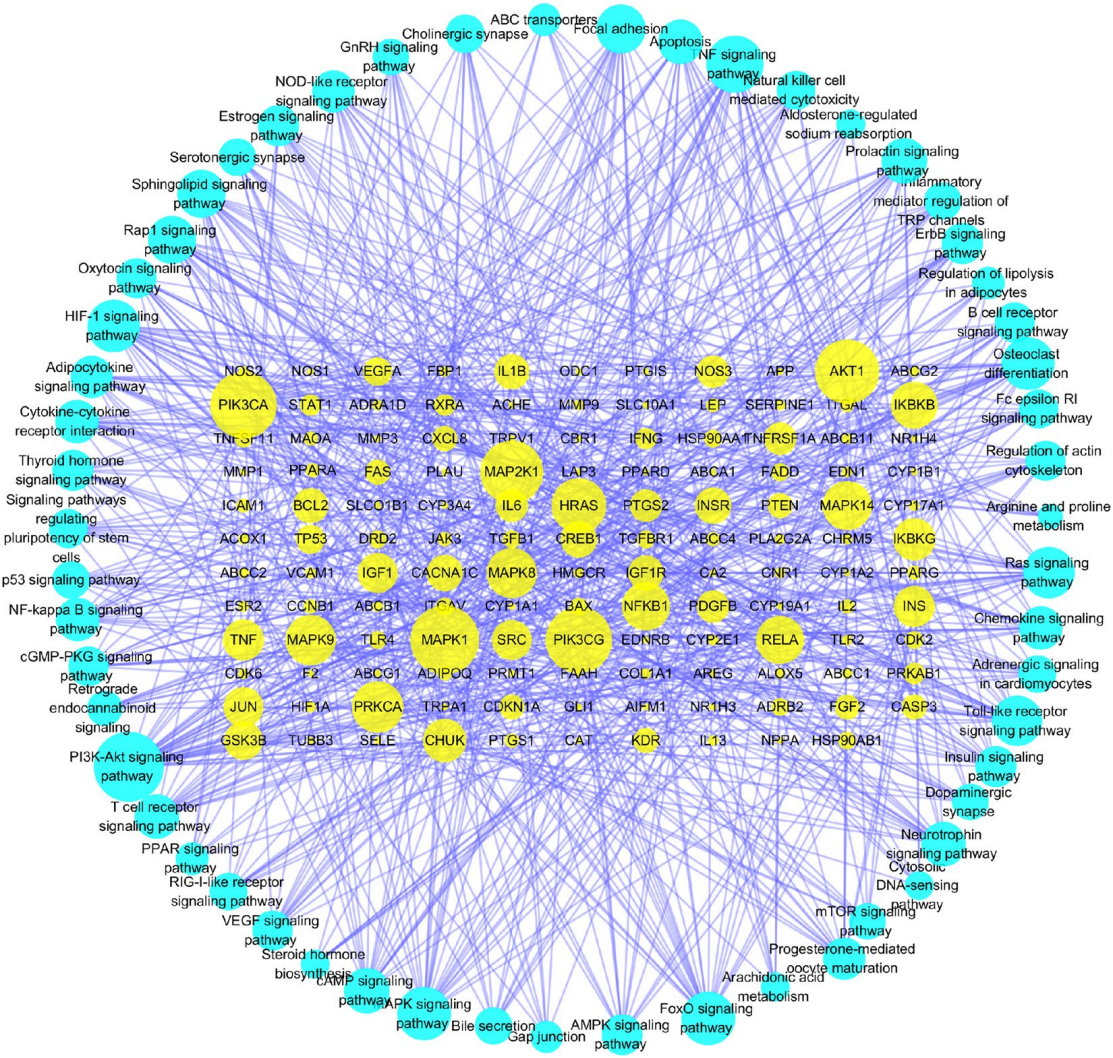
Non-disease associated Target-Pathway (T-P) network of Shenzhu Capsule. Nodes represent targets and non-disease associated pathways. The node size is related to the degree of nodes.

**Figure 7:**
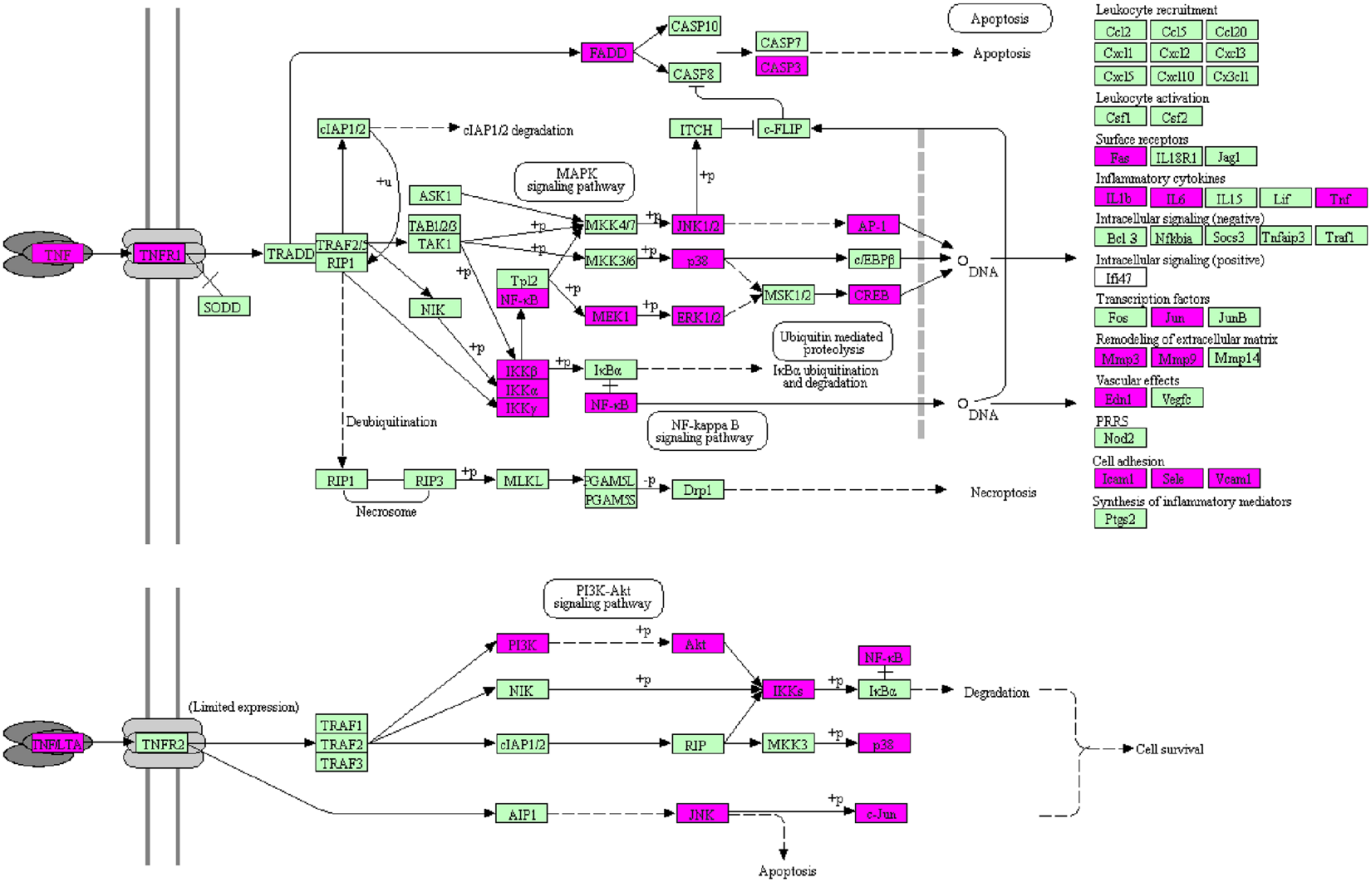
Distribution of the targets of Shenzhu Capsule on the compressed TNF signaling pathway. The red nodes are potential targets of SZC, and the light blue nodes are relevant targets in the pathway. The compressed signaling pathway was obtained from KEGG^76^.

Pathway analysis plays an important role in system pharmacology as it bridges the gaps between receptor-ligand interactions and pharmacodynamic outputs. Because enriched disease pathways could interfere with the correct judgment^16^, only non-disease associated pathways were used to construct T-P network. By KEGG pathway analysis integrated in DAVID, all the targets and corresponding pathways were obtained (Supplementary Table S6), and then mapped into T-P network by Cytoscape 3.5.1 (Fig. 6). The T-P network showed 806 interactions between 131 targets and 55 non-disease associated pathways with *P* < 0.01. Of these pathways, PI3K-Akt signaling pathway (degree = 39) had the biggest number of connections with the targets, followed by TNF signaling pathway (degree = 29), MAPK signaling pathway (degree = 25) and FoxO signaling pathway (degree = 25). The roles of these high-degree pathways in UC have been well established^35–37^. Besides, some other pathways also participated in the development of UC, such as NF-*κ*B signaling pathway, PPAR signaling pathway, Toll-like receptor signaling pathway^38–40^. An example of the synergism of compounds that act on TNF signaling pathway to treat UC is shown in Fig. 7. In addition to the non-disease associated pathways, SZC can also influence many disease associated pathways, including pathways in cancer (degree = 50) and colorectal cancer (degree = 15). Because UC has the potential to develop into colorectal cancer^41^, it can be deducted that SZC could also prevent the development of UC to more severe colorectal cancer. Another important disease associated pathway of SZC is inflammatory bowel disease pathway. Supplementary Fig. S3 shows the specific targets of SZC in this disease associated pathway. Taken together, the results indicated that SZC could exert its therapeutic effects through influencing multiple pathways and acting on multiple targets in each pathway.

### Pathway comparison of SZC and synthetic drugs

KEGG pathway analysis of synthetic drugs showed that 25 targets were involved in 12 non-disease associated pathways with *P* < 0.01 (Supplementary Table S7). On the contrary, SZC can influence 55 non-disease associated pathways as aforementioned. Comparing of these pathways showed that synthetic drugs and SZC share 9 pathways while 3 pathways are exclusive to synthetic drugs (Fig. 4B). For the 9 shared pathways, SZC could act on more targets than synthetic drugs in each pathway (Fig. 4C). Taking TNF signaling pathway as an example, SZC can act on 34 targets in this pathway (Fig. 7), while synthetic drugs can act on 13 targets in this pathway (Supplementary Fig. S4). Therefore, SZC could act on more pathways and targets in the pathways, which strongly supported the holistic and synergistic properties of TCM.

In addition, for the synthetic drugs, the pathways such as osteoclast differentiation, antigen processing and presentation, intestinal immune network for IgA production, TNF signaling pathway, NF-*κ*B signaling pathway are mainly associated with immune responses and inflammatory reactions. On the contrary, SZC can influence 131 targets and 55 non-disease associated pathways (Supplementary Table S6). These pathways are involved in not only immune responses and inflammatory reactions (such as TNF signaling pathway, toll-like receptor signaling pathway, osteoclast differentiation), but also a great number of pathways of wide range of functions (such as FoxO signaling pathway which is associated with apoptosis, cell-cycle control, glucose metabolism, oxidative stress resistance, and longevity) and some special functional pathways (such as adipocytokine signaling pathway which is highly associated with production of adiponectin). Noteworthy, adiponectin has been proved to be a protective factor for UC^42,43^. Taken together, by comparing pathways of SZC and synthetic drugs, we can conclude that they share similarities in regulating pathways related to immune and inflammatory reactions, and the unique feature of SZC is that it can influence many pathways of wide range of functions and pathways of special protective function.

## Discussion

For synthetic drugs, they are conventionally designed to exert their therapeutic effects through targeting a special target with one compound (one target, one drug paradigm)^44,45^. In past decades, with the prevalence of chronic and complex diseases, clinical strategy for treatment of diseases has begun to embrace the idea of combination of different medicinal compounds in treating diseases such as AIDS, diabetes, idiopathic pulmonary fibrosis^46–48^. In this background, to reveal the mechanism feature of SZC in comparison of synthetic drugs, we viewed synthetic drugs as a whole. Meanwhile, to achieve this goal, a system pharmacology-based approach including active screening, targets prediction, GO and DAVID enrichment analysis was used. Although it is not possible to administrate all the synthetic drugs listed in our study to a patient in clinic, by viewing synthetic drugs as a whole, it enables us to better understand the mechanism feature of SZC in treating UC, and to better understand the mechanism differences between SZC and synthetic drugs.

Chemical space stands for all molecules that might exist and could be used as the basis of virtual screening^49^. In our study, PCA was adopted to compare the distribution of SZC compounds and synthetic drugs in chemical space. Results showed that the area of compounds in SZC encompasses the area of synthetic drugs (Fig. 2), indicating that some compounds in SZC share similar chemical properties with synthetic drugs and they might have similar biological properties. Some other studies also compared the distribution of synthetic drugs with herbal products in chemical space. Our result is in line with another study although the distribution of synthetic drugs in their study is more scattered^50^. Yet, there are some studies showed different results. One study showed that nature products and synthetic drugs share similar chemical space^51^. Another two studies showed that nature products and synthetic drugs share some chemical space but both with some unique chemical space^52,53^. Compared with those studies, the distribution of synthetic drugs in our study were more concentrated and the area is much narrower. This difference might be attributed to the fact that only medicines used for treatment of UC were applied to perform PCA while the other studies used all synthetic drugs in databases.

TCM theory holds that UC is caused by deficiency of Spleen and Qi, and the principle for treatment of UC should be based on supplement of Spleen and Qi^54^. SZC is an TCM that is used to treat UC by supplement of Spleen and Qi. It contains two herbal medicines, Renshen and Baizhu, which are typical herbs of the effects to invigorate Spleen and tonify Qi^55,56^. Studies have shown that Spleen deficiency and Qi deficiency could result in increased intestinal activity of IL-2, Il-6, INF-*γ*, TNF-*α*^57–59^. In our study, we found out that compounds such as atractylenolide I, dianthramine, paeonol could act on IL-6, TNF-α, INF-*γ*, etc. These targets are important molecules in immune and inflammatory related pathways such as TNF signaling pathway, Toll-like receptor signaling pathway, NF-*κ*B signaling pathway. Therefore, SZC might exert its curative effects through compounds such as atractylenolide I, dianthramine, paeonol, etc., acting on targets such as IL-6, TNF-*α*, INF-*γ*, etc., in pathways such as TNF signaling pathway, Toll-like receptor signaling pathway, NF-*κ*B signaling pathway to invigorate Spleen and Qi.

TCM prescriptions usually contain numerous ingredients that synergistically and holistically act on the diseases^14,60^. In our study, very common compounds such as *α*-amyrin, *β*-sitosterol and γ-selinene are found to be active in treating UC (Supplementary Table S3). For those compounds, they might be neglected by traditional *in vivo* or *in vitro* experiment-based pharmacology. But for system pharmacology, all the compounds were equally screened by same parameters to obtain the effective compounds, thus the traditionally neglected compounds could be screened. This demonstrated the advantage of system pharmacology in screening effective compounds. Besides, some of these common compounds such as *α*-amyrin and *β*-sitosterol have been demonstrated to be able to attenuate dextran sulfate sodium-induced colitis and TNBS-induced colitis, respectively^18,25^. These studies demonstrated that systems pharmacology is adoptable to screen the effective compounds.

Gut microbiota is an important factor in UC development^61^. One interesting thing in our study is that, although *α*-longipinene (C16 in Supplementary Table S1) is not bioactive on human body according to our screen parameters, it has the ability to inhibit *Candida albicans*^62^, one kind of microorganism in colon that can augment colitis inflammation^63^. Therefore, SZC can ameliorate UC by acting directly on human body and indirectly on gut microbiota. In addition, both Renshen and Baizhu contain large amount of polysaccharides, which are known to play important role in shaping gut microbiota^64^. Therefore, except for the small molecular compounds, the polysaccharides in SZC might be responsible for curative effects of SZC in treatment of UC by modulation of gut microbiota. For the reasons above, we think that more attentions should be paid to study the effects of SZC on gut microbiota.

With the adoption of systems pharmacology, the unique mechanism features of SZC in comparison with synthetic drugs were obtained. The first feature is associated with the number and category of targets. Comparison of targets showed that SZC can act on more targets than synthetic drugs. Specifically, 191 targets only belong to SZC, 35 targets only belong to synthetic drugs, and 19 targets are shared by SZC and synthetic drugs (Fig. 4A). The interesting finding about the target of SZC is that, different from synthetic drugs, the compounds in SZC not only targeted on key modulators involved in UC, but also on other targets related to UC complications such as bloody diarrhea (F2, thrombin). The second feature of SZC is linked to the number and type of pathways. Comparison of pathways showed that SZC can act on more pathways than synthetic drugs. Specifically, SZC can influence 55 non-disease associated pathways, synthetic drugs can influence 12 non-disease associated pathways, and 9 non-disease associated pathways are shared by SZC and synthetic drugs (Fig. 4B). And for the types of pathways, SZC could not only regulate pathways related to immune and inflammatory reactions but also act on pathways of special or wide range of functions such as FoxO signaling pathway. On the contrary, the mechanism feature of synthetic drugs is that they act mainly on a few of pathways that are associated with immune and inflammatory reactions.

Another important feature of SZC is related to the enriched disease associated pathways. In our study, KEGG pathway analysis generated 3 important disease associated pathways about SZC, including cancer pathway, colorectal cancer pathway, and inflammatory bowel disease pathway. Considering that UC is classified under the category of inflammatory bowel disease and SZC can target directly on inflammatory bowel disease pathway, it is reasonable to believe that SZC can play its therapeutic role by directly acting on UC related targets. In clinic, UC is also associated with a predisposition to develop to colorectal cancer^3^. Thus, SCZ may also play a preventive role in UC patients by preventing UC to develop to more severe colorectal cancer. Therefore, SZC not only plays a direct curative role in treatment of UC but also an indirect preventive role to prevent UC to develop to colorectal cancer.

In our study, there are still some drawbacks that should be noted. Although some targets of SZC have been confirmed by literatures and molecular docking, many predicated targets are still needed to be validated by *in vivo* or i*n vitro* experiments. Another fact is related to the shortcomings of system pharmacology to reveal the mechanisms of SZC. Firstly, different dose might lead to different pharmacological effects, this approach needs more accurate algorithm to obtain that potential dose-effect outcome. Secondly, not all compounds from Baizhu and Renshen are currently known, and so do not the targets of UC, correspondingly, important bioactive substances and targets may be missing from the databases and literatures. Thirdly, even all targets are known, this *in silico* approach might not obtain targets that will appear in real *in vivo* or i*n vitro* condition because of the drawbacks of algorithm. Last but not least, some metabolites might be bioactive but not all the metabolites are currently known, and thus not all the targets can be predicted based on the structure of metabolites. Nevertheless, as a new tool to systemically study the mechanisms of herbal medicines, it enables us to discover bioactive ingredients and drug targets in an efficient way.

## Conclusion

Both synthetic drugs and TCMs are used to treat UC in China. In our study, we presented a strategy to obtain the mechanism and mechanism feature of SZC in treatment of UC by comparing chemical space, targets, pathways of SZC and synthetic drugs. By bioactive compounds screening, 73 bioactive compounds in SZC including atractylenolide I, atractylenolide II, atractylone, β-caryophyllene, etc., were obtained. Then, the chemical space distribution of SZC and synthetic drugs was compared by PCA analysis, and it showed that the chemical space of SZC covers the space of synthetic drugs. By target fishing, the targets of SZC were obtained. And then, the mechanism features of SZC were obtained by comparing the targets and pathways of SZC and synthetic drugs. We found out that SZC can act on more targets and pathways than synthetic drugs. In addition, SZC can not only regulate immune and inflammatory reactions but also act on ulcerative colitis complications and prevent UC to develop into colorectal cancer whereas synthetic drugs mainly regulate immune and inflammatory reactions. The results demonstrated the multitargeting nature of SZC, and we believe that the strategy can be helpful for understanding the difference between TCM and synthetic drugs.

## Methods

### Chemical compounds retrieving of SZC and synthetic drugs

All the constituents and molecular structures of Renshen (*Ginseng radix et rhizoma*, the dried roots of *Panax ginseng* C.A. Mey.) and Baizhu (*Atractylodis macrocephalae rhizoma*, the dried roots of *Atractylodes macrocephala* Koidez.) were retrieved and downloaded from Traditional Chinese Medicine Systems Pharmacology Database and Analysis Platform (TCMSP, http://ibts.hkbu.edu.hk/LSP/tcmsp.php), a professional system pharmacology platform that is designed for Chinese herbal medicines^65^, and then supplemented manually with text-mining method including Google Scholar and PubMed. Because glycosides might be deglycosylated in intestinal tract by gut microbiota, aglycones were also incorporated into the compound library. The synthetic drugs and corresponding parameters that used for PCA were retrieved from DrugBank (http://www.drugbank.ca/), in this process, the drugs that belong to protein-based therapies or total herbal extracts were excluded.

### Screening active compounds of SZC (ADME screening)

To screen the potential active compounds from TCM, previous prediction is an indispensable step in drug development and in determining the therapeutic mechanisms of TCM formula. The bioactive compounds in SZC were obtained by two important parameters including bioavailability (OB) and drug-likeness (DL). OB, which is an important pharmacokinetic parameter in screening active compounds for orally-administrated drugs, represents the percentage of the orally-administered dose that arrives in human body unchanged^66^. DL is related to factors that influence pharmacodynamics and pharmacokinetics of molecules including absorption, distribution, metabolism, and excretion (ADME)^67^. An in-house tool Obioavail1.1 system that integrates the metabolism (P450 3A4) and transport (P-glycoprotein) information was applied to get the OB (Xu *et al*.^68^). Database-dependent DL was evaluated by Tanimoto similarity which is defined as T(*x*, *y*) = (*x* × *y*)/(|*x*|^2^ + |*y*|^2^ − *x* × *y*), where *x* represents the molecular descriptors in SZC and *y* represents the average DL index of molecules in DrugBank^68^. In our study, compounds with OB ≥30% and DL ≥0.18 were selected as candidate active compounds for further analysis^65^. Considering some compounds that did not pass the screening parameters might exhibit profound pharmacological effects and high contents, those compounds that reportedly exhibit strong pharmacological effects yet with low OB or DL were also included.

### Principal component analysis (PCA) of SZC and synthetic drugs

PCA, a compound feature mapping method, was employed to visualize all active compounds in SZC and synthetic drugs deposited in DrugBank that can treat UC. Noteworthy, only those small molecular synthetic drugs made up of single effective compound were included. PCA was applied to visualize the distribution of those compounds by submitting four parameters characteristic of drug-related physicochemical properties including MW, Clogp, nHDon, and nHAcc to SIMCAP + software (version 14.1, Umetris)^14^.

### Targets prediction of SZC

In order to identify the targets of active compounds in SZC, a comprehensive approach including chemometric method and information integration were applied. First, the active compounds were submitted to various on-line servers or databases viz. Bioinformatics Analysis Tool for Molecular Mechanism of Traditional Chinese Medicine (BATMAN-TCM, http://bionet.ncpsb.org/batman-tcm/index.php/Home/Index/index)^69^, Similarity Ensemble Approach (SEA, http://sea.bkslab.org)^70^, TCM-Mesh (http://mesh.tcm.microbioinformatics.org/)^15^, PhID (http://phid.ditad.org/MetaNet/)^71^, TCMSP (http://ibts.hkbu.edu.hk/LSP/tcmsp.php)^65^, Therapeutic Targets Database (TTD, http://bidd.nus.edu.sg/group/ttd/)^72^, BindingDB database (http://www.bindingdb.org/bind/index.jsp)^73^. Then, the genes and proteins related to UC were retrieved from Combinatory Drug Discover Platform (http://ibts.hkbu.edu.hk/SDF/index.php), Comparative Toxicogenomics Database (CTD, http://ctdbase.org/)^74^ and only those genes with interference score ≥30 were preserved. Known therapeutic targets of synthetic drugs were obtained from TTD and DrugBank^8^. Only the targets of Homo sapiens origin were kept for further analysis in the whole process.

### Compound-target interaction validation by molecular docking

Molecular docking is a widely used tool to validate the targets of compounds screened by system pharmacology^75^. The 3D structure of compounds in SZC were collected from TCMSP, and the crystal structure of targets were downloaded from Protein Data Bank (http://www.rcsb.org/) with PDB file format. Before docking, the solvent molecules in targets were removed and the structure of targets were optimized by merging nonpolar hydrogens with Gasteiger charges, and all the rotatable bonds in ligands were applied with torsion angles. Then, the targets and ligands were saved as PDBQT format. AutoDock 4.2.6 was applied to perform molecular docking with the conformations of targets and ligands set as rigid and flexible, respectively. 60 Å × 60 Å × 60 Å 3D grids was centered around the known ligand binding sites, and Lamarckian genetic algorithm was used to optimize the conformation. The rest parameters were set to default. After docking, the conformation with the lowest binding energy was selected and viewed by PyMOL.

### Gene ontology (GO) analysis

Biological process (BP), molecular function (MF), and cellular component (CC)of GO analysis was applied to determine the biological, molecular and cellular properties of target genes. Webserver the Database for Annotation, Visualization and Integrated Discovery (DAVID, https://david.ncifcrf.gov/home.jsp) v6.8 were applied to perform GO enrichment analysis for the genes targeted by SZC. In our research, GO terms with *P* value < 0.01 were used.

### Network construction

In order to understand the complicate interactions between the active compounds in SZC and their corresponding targets, visualized networks were constructed, including Compound-Target network (C-T network) and Target-Pathway network (T-P network). In C-T network, nodes represent compounds or corresponding targets and edges stand for connections between compounds and targets. In T-P network, the nodes stand for compound targets or corresponding pathways the targets enrolled in. The pathway information was extracted from KEGG (Kyoto Encyclopedia of Genes and Genomes, http://www.kegg.jp), and only the pathways of *P* < 0.01 were preserved^76^. The compressed signaling pathway figures were obtained and revised from KEGG as well^76^. As enriched disease pathways could interfere with the correct judgment^16^, only non-disease associated pathways were used to construct T-P network. Both visualized networks were generated by Cytoscape 3.5.1 (http://www.cytoscape.org/), a general platform for complex network analysis and visualization.

## Electronic supplementary material

Supplementary figures and tables
