# Supplementary material for: Systems pharmacology reveals the unique mechanism features of Shenzhu Capsule for treatment of ulcerative colitis in comparison with synthetic drugs
Source: Sci Rep. 2018 Nov 1;8:16160. doi: 10.1038/s41598-018-34509-1 (PMC6212405; doi:10.1038/s41598-018-34509-1)
Supplement: Supplementary file 1 — Supplementary figures and tables [file 41598_2018_34509_MOESM1_ESM.pdf]

Systems pharmacology reveals the unique mechanism features of Shenzhu Capsule for treatment of ulcerative colitis in comparison with synthetic drugs

Wuwen Feng[1,†], Hui Ao[1,†], Shijun Yue[2], Cheng Peng[1,3]

[1]School of Pharmacy, Chengdu University of Traditional Chinese Medicine, Chengdu, China. [2]Key Laboratory of Marine Drugs (Ministry of Education of China), School of Medicine and Pharmacy, Ocean University of China, Qingdao, China. [3]State Key Laboratory Breeding Base of Systematic Research, Development and Utilization of Chinese Medicine Resources, Chengdu University of Traditional Chinese Medicine, Chengdu, China.

[†] These authors contributed equally to this work.

Correspondence should be addressed to C.P. (pengchengcxy@126.com)

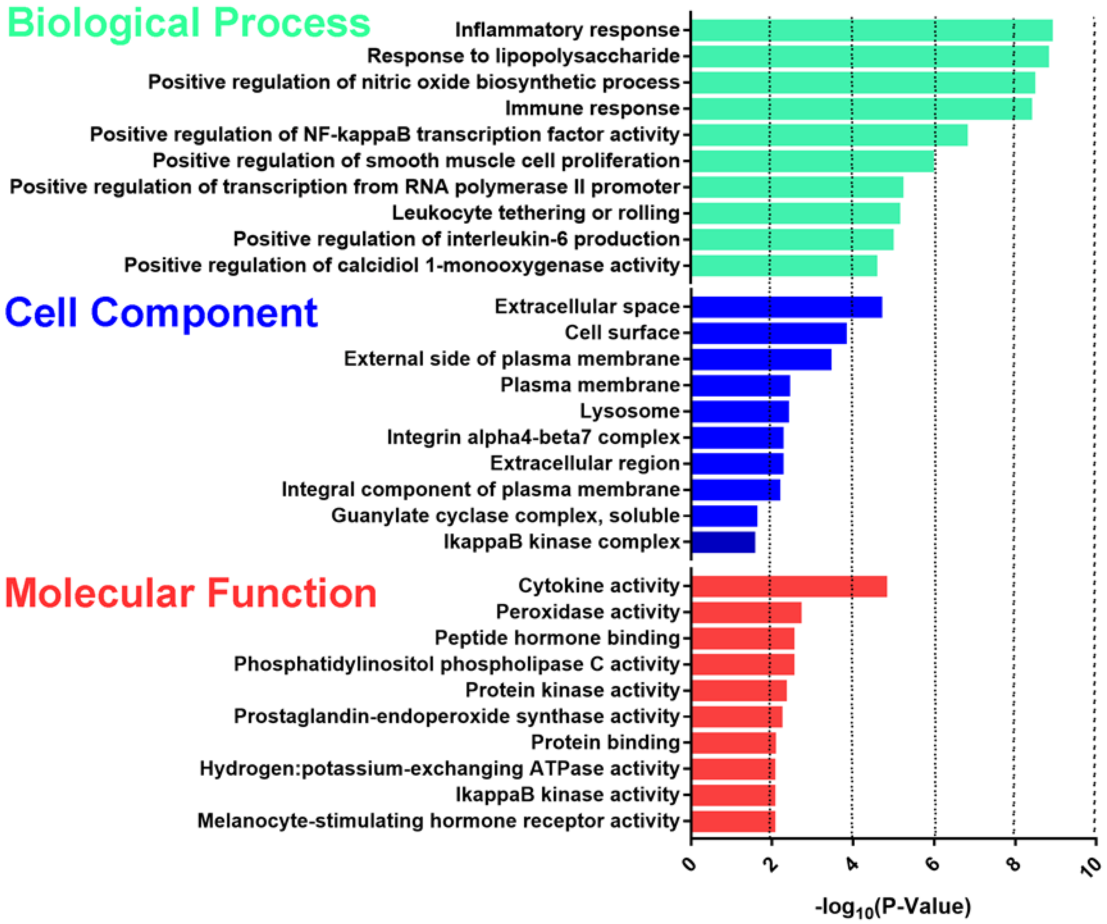

Supplementary Figure S1 Gene ontology (GO) enrichment analysis of synthetic drugs used for treatment of ulcerative colitis. The *y*-axis represents biological process (BP), cell component (CC) and molecular function (MF) terms of target genes, and the *x*-axis stands for $-\log_{10}(P\text{-Value})$.

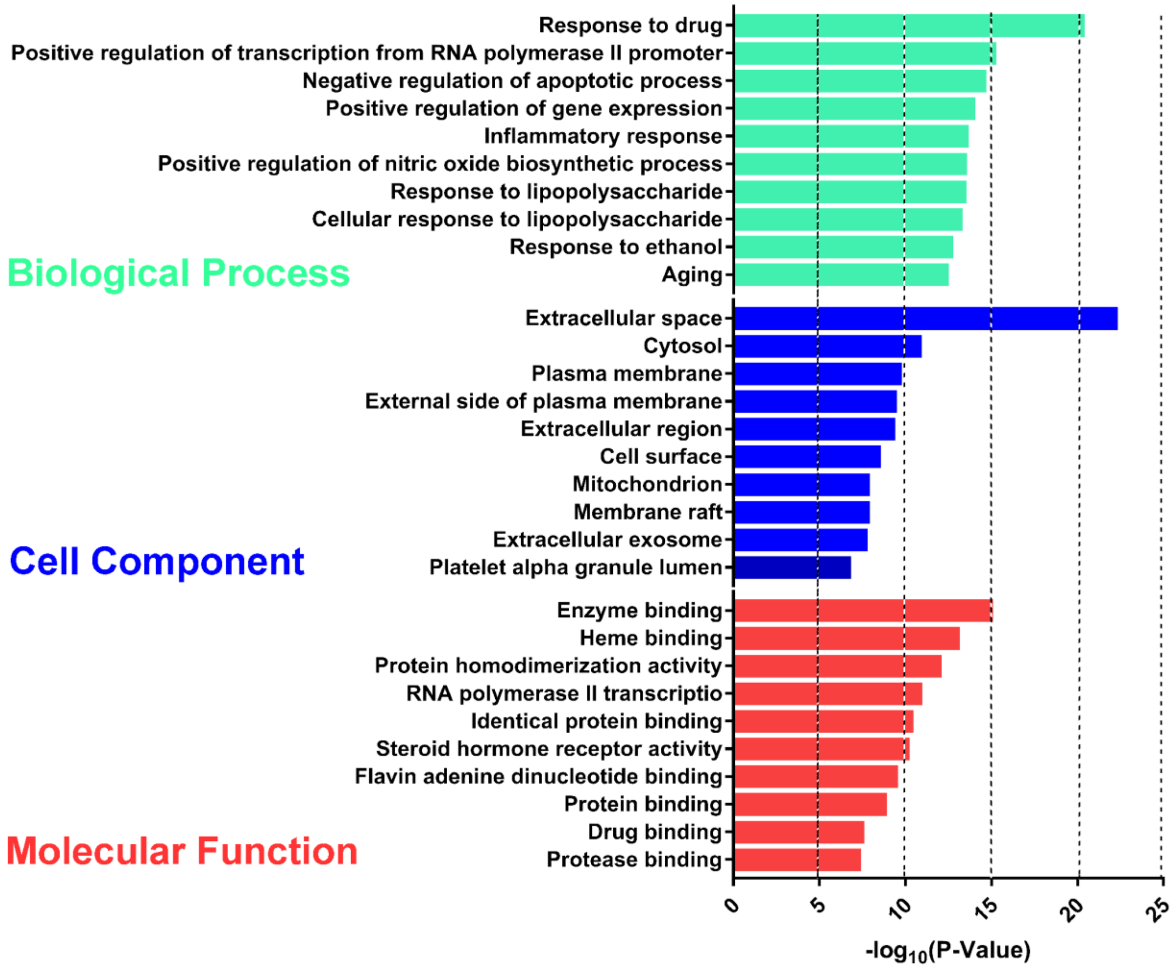

Supplementary Figure 2 Gene ontology (GO) enrichment analysis of ShenZhu Capsule. The *y*-axis represents biological process (BP), cell component (CC) and molecular function (MF) terms of target genes, and the *x*-axis stand for $-\log_{10}$(*P*-Value).

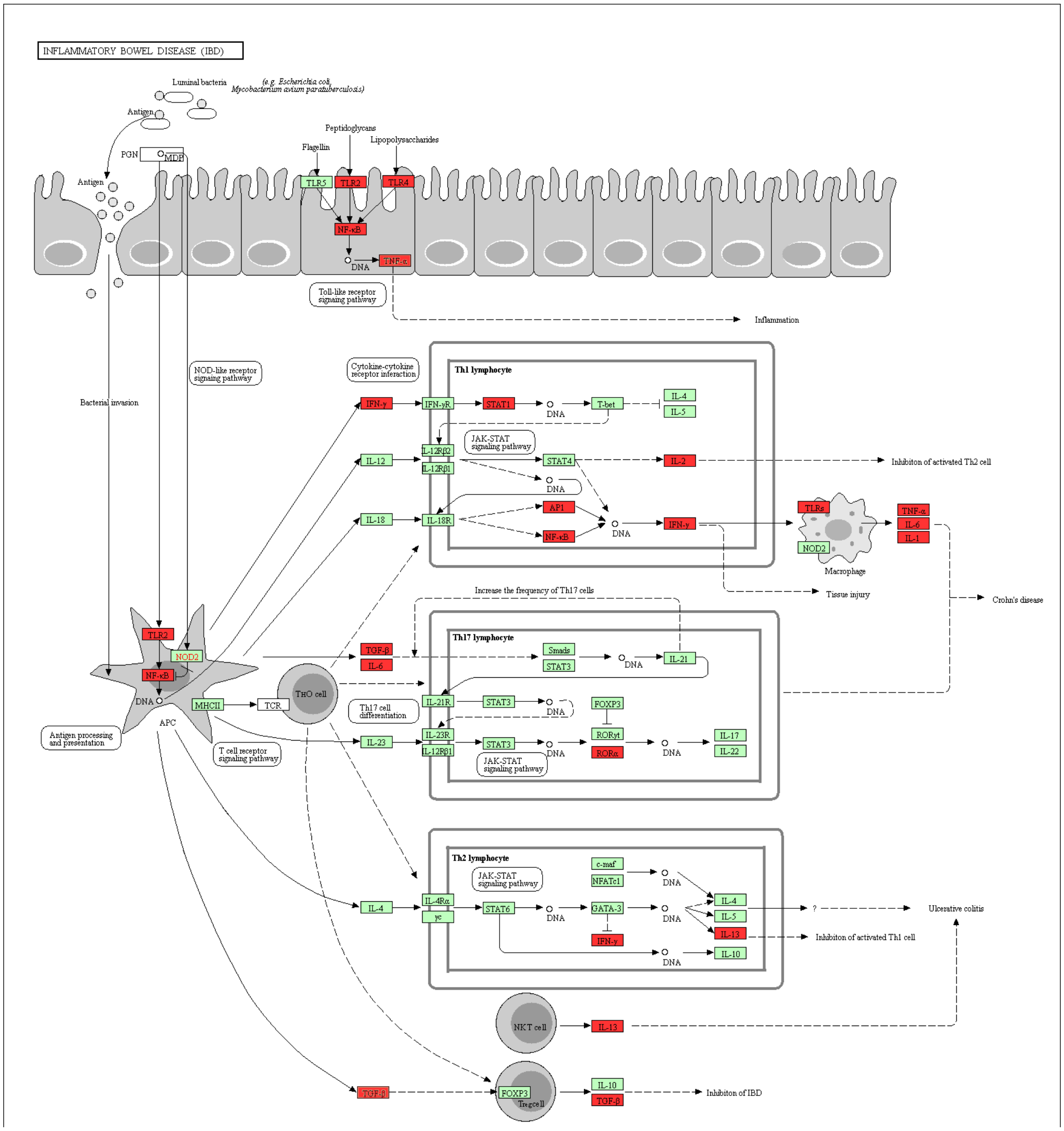

Supplementary Figure S3 Distribution of targets of Shenzhu Capsule on the compressed inflammatory bowel disease (IBD) pathway. The red nodes are potential targets of SZC, and the light blue nodes are relevant targets in the pathway. The compressed signaling pathway was obtained from KEGG[1].

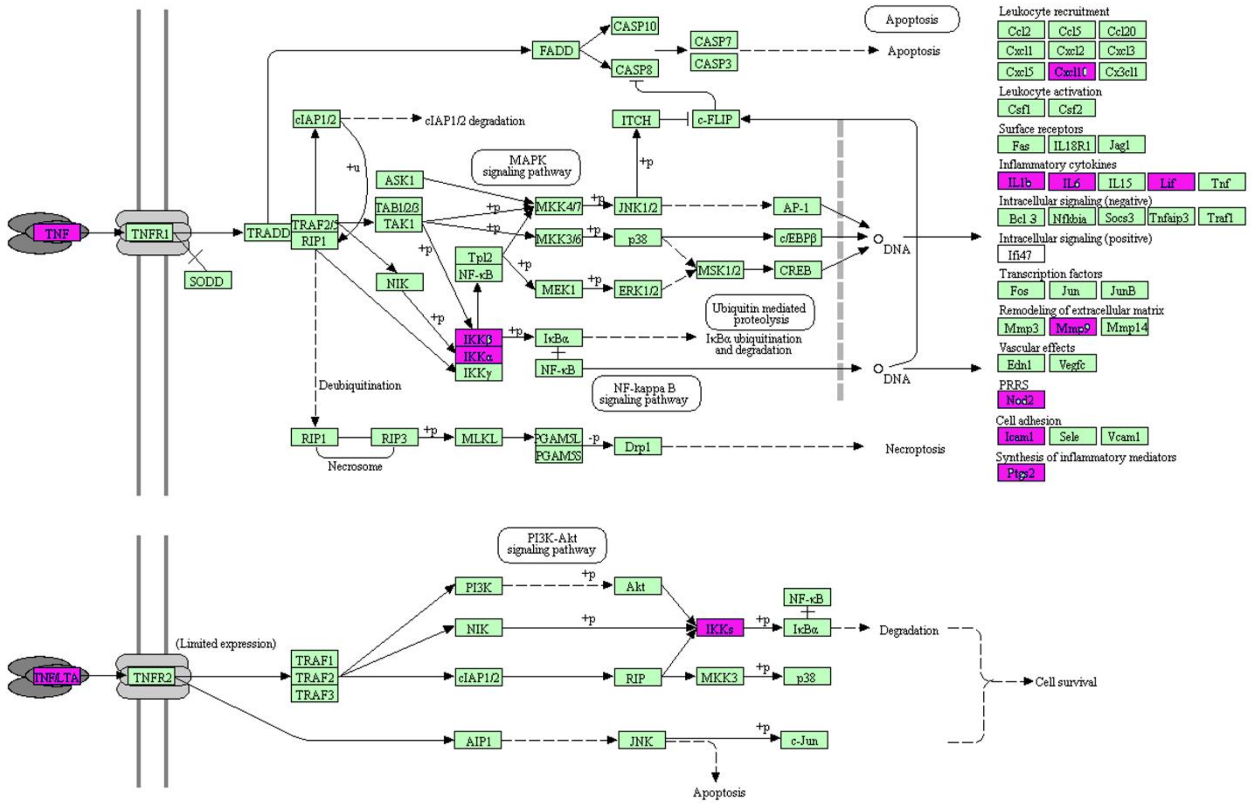

Supplementary Figure S4 Distribution of the targets of synthetic drugs on the compressed TNF signaling pathway. The red nodes are potential targets of synthetic drugs, and the light blue nodes are relevant targets in the pathway. The compressed signaling pathway was obtained from KEGG[1].

Reference:

1 Kanehisa, M., Furumichi, M., Tanabe, M., Sato, Y., & Morishima, K. KEGG: new perspectives on genomes, pathways, diseases and drugs. Nucleic Acids Res. 45, D353-D361 (2017).

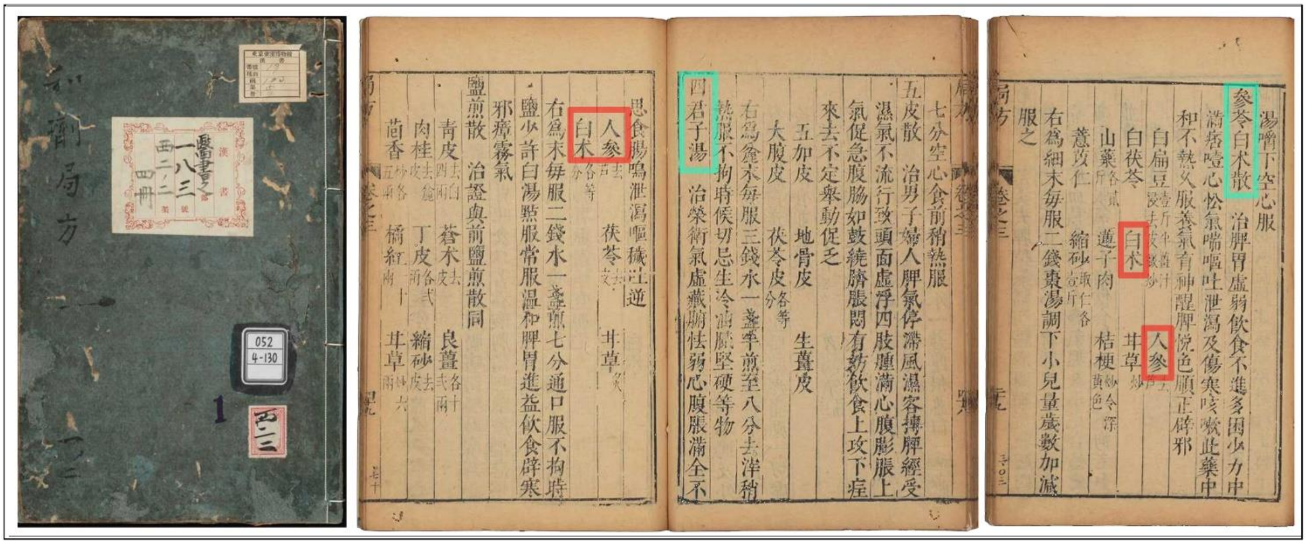

Supplementary Figure S5 Renshen (人参) and Baizhu (白术) recorded in ancient Chinese formulae. (Pictures were downloaded from http://zhongyibook.com/taipinghuiminhejijufang-scan/146.html, and http://zhongyibook.com/taipinghuiminhejijufang-scan/166.html)

Supplementary Table S1 The detailed information of all compounds in Renshen and Baizhu.

Compound No.	herb	Compound	CAS Number	MW	ClogP	Hdon	Hacc	OB	Caco-2	DL
C1	*A. macrocephala*	(2S)-2-azaniumyl-3-(4-hydroxyphenyl)propanoate	60-18-4	181.21	0.69	4	4	57.55	-0.1	0.05
C2	*A. macrocephala*	3*β*-acetoxyatractylone	61206-10-8	274.39	3.39	0	3	54.07	1.13	0.22
C3	*A. macrocephala*	*α*-amyrin	638-95-9	426.8	7.35	1	1	39.51	1.42	0.76
C4	*A. macrocephala*	atractylenolide I	73069-13-3	230.33	3.32	0	2	37.37	1.3	0.15
C5	*A. macrocephala*	ethyl pivaloylacetate	17094-34-7	172.25	1.69	0	3	40.52	0.82	0.03
C6	*A. macrocephala*	L-lysin	56-87-1	146.22	-0.68	5	4	29.33	-0.66	0.02
C7	*A. macrocephala*	(3S)-3-[(1R)-1,5-dimethylhex-4-enyl]-6-methylenecyclohexene	20307-83-9	204.39	5.14	0	0	19.86	1.88	0.06
C8	*A. macrocephala*	8β-ethoxy atractylenolide III	N/A	276.41	3.68	0	3	35.95	1.08	0.21
C9	*A. macrocephala*	uridine	58-96-8	244.23	-2.45	4	8	10.49	-1.14	0.11
C10	*A. macrocephala*	juniper camphor	N/A	222.41	3.93	1	1	33.3	1.44	0.1
C11	*A. macrocephala*	(3S)-3-azaniumyl-4-hydroxy-4-oxobutanoate	56-84-8	133.12	-1.25	4	5	79.74	-1.02	0.02
C12	*A. macrocephala*	selina-4(14),7(11)-dien-8-one	54707-47-0	218.37	3.81	0	1	32.31	1.42	0.1
C13	*A. macrocephala*	(1R)-2-methyl-1-phenylprop-2-en-1-ol	N/A	148.22	2.32	1	1	75.1	1.27	0.03
C14	*A. macrocephala*	(24S)-24-propylcholesta-5-ene-3β-ol	64997-52-0	428.82	8.54	1	1	36.23	1.45	0.78
C15	*A. macrocephala*	(+/-)-isoborneol	124-76-5	154.28	1.98	1	1	86.98	1.27	0.05

C16	*A. macrocephala*	*α*-longipinene	5989/8/2	204.39	4.12	0	0	53.26	1.83	0.12
C17	*A. macrocephala*	prolinum	147-85-3	115.15	-0.06	2	3	77.57	0.22	0.01
C18	*A. macrocephala*	scopoletol	92-61-5	192.18	1.62	1	4	27.77	0.71	0.08
C19	*A. macrocephala*	biatractylolide	N/A	462.68	6.68	0	4	17.45	0.83	0.81
C20	*A. macrocephala*	stigmast-22E-en-3*β*-ol	481-18-5	414.79	7.89	1	1	10.39	1.43	0.75
C21	*A. macrocephala*	atractylone	6989-21-5	216.35	4.11	0	1	41.1	1.76	0.13
C22	*A. macrocephala*	akridin	260-94-6	179.23	3.35	0	1	33.71	1.63	0.1
C23	*A. macrocephala*	2-[(2R,5S,6S)-6,10-dimethylspiro-[4.5]dec-9-en-2-yl]-propan-2-ol	23811-08-7	222.41	3.67	1	1	38.59	1.34	0.09
C24	*A. macrocephala*	istidina	71-00-1	155.18	-1.01	4	4	53.18	-0.25	0.03
C25	*A. macrocephala*	α-humulene	6753-98-6	204.39	5.04	0	0	22.98	1.88	0.06
C26	*A. macrocephala*	L-valin	72-18-4	117.17	0.24	3	3	53.33	0.04	0.01
C27	*A. macrocephala*	*α*-curcumene	4176-17-4	202.37	5.34	0	0	4.68	1.93	0.06
C28	*A. macrocephala*	(1S,2R,4R)-neoiso-dihydrocarveol	51773-45-6	154.28	2.58	1	1	52.4	1.38	0.03
C29	*A. macrocephala*	bis(2-methylpropyl) benzene-1,2-dicarboxylate	84-69-5	278.38	3.92	0	4	49.63	0.85	0.13
C30	*A. macrocephala*	14-acetyl-12-senecioyl-2E,8E,10E-atractylentriol	113269-37-7	355.44	3.21	0	5	60.31	0.33	0.31
C31	*A. macrocephala*	gulutamine	56-86-0	147.15	-0.92	4	5	6.66	-1.05	0.02
C32	*A. macrocephala*	phenylalanine	63-91-2	165.21	0.96	3	3	41.62	0.36	0.04
C33	*A. macrocephala*	*D*-serin	56-45-1	105.11	-1.49	4	4	83.59	-0.94	0.01
C34	*A. macrocephala*	(5*E*,9*Z*)-3,6,10-trimethyl-4,7,8,11-	19912-61-9	216.35	4.63	0	1	43.17	1.77	0.1

		tetrahydrocyclodeca-[b]-furan								
C35	*A. macrocephala*	*β*-eudesmol	473-15-4	222.41	3.72	1	1	26.09	1.32	0.1
C36	*A. macrocephala*	(2S)-2-amino-5-(diaminomethylideneazaniumyl)pentanoate	74-79-3	174.24	-1.11	7	6	47.64	-0.49	0.03
C37	*A. macrocephala*	hemo-sol	5989-27-5	136.26	3.5	0	0	39.84	1.83	0.02
C38	*A. macrocephala*	12-senecioyl-2*E*,8*E*,10*E*-atractylentriol	113269-39-9	312.39	2.5	0	4	62.4	0.01	0.22
C39	*A. macrocephala*	methose	57-48-7	180.18	-2.69	5	6	1.68	-1.8	0.03
C40	*A. macrocephala*	polymannose	30142-85-9	180.18	-2.68	5	6	1.76	-1.94	0.03
C41	*A. macrocephala*	atractylenolide II	73069-14-4	232.35	3.57	0	2	47.5	1.3	0.15
C42	*A. macrocephala*	2-[(1R,3S,4S)-3-isopropenyl-4-methyl-4-vinylcyclohexyl]-propan-2-ol	639-99-6	222.41	3.7	1	1	19.03	1.37	0.07
C43	*A. macrocephala*	atractylenolide III	73030-71-4	248.35	2.93	1	3	68.11	0.75	0.17
C44	*A. macrocephala*	*D*-camphene	5794/3/6	136.26	2.93	0	0	34.98	1.81	0.04
C45	*A. macrocephala*	*L*-Ile	73-32-5	131.2	0.7	3	3	59.05	0.06	0.02
C46	*A. macrocephala*	*γ*-elemene	29873-99-2	204.39	4.93	0	0	23.79	1.87	0.06
C47	*A. macrocephala & P. ginseng*	*β*-humulene	116-04-1	204.39	5.09	0	0	26.87	1.82	0.06
C48	*A. macrocephala & P. ginseng*	alloaromadedrene	25246-27-9	204.39	4.22	0	0	53.46	1.83	0.1

C49	*A. macrocephala & P. ginseng*	palmitic acid	67701-02-4	256.48	6.37	1	2	19.3	1.09	0.1
C50	*A. macrocephala & P. ginseng*	*β*-caryophyllene	87-44-5	204.39	4.75	0	0	29.7	1.83	0.09
C51	*A. macrocephala & P. ginseng*	*β*-selinene	17066-67-0	204.39	4.81	0	0	24.39	1.83	0.08
C52	*P. ginseng*	*n*-heptadecanol	52783-44-5	256.53	6.9	1	1	12.97	1.31	0.09
C53	*P. ginseng*	ginsenoside Rh5	N/A	636.96	2.79	7	9	3.23	-1.36	0.57
C54	*P. ginseng*	ginsenoside Re	51542-56-4	947.3	0.27	12	18	4.27	-3.2	0.12
C55	*P. ginseng*	2,3,8-trimethyldecane	62238-14-6	184.41	5.69	0	0	5.51	1.79	0.03
C56	*P. ginseng*	*p*-glucosyloxymandelonitrile	N/A	298.37	-0.47	5	6	12.12	-0.92	0.18
C57	*P. ginseng*	methyl pentadecanoate	7132-64-1	256.48	6.16	0	2	18.82	1.37	0.1
C58	*P. ginseng*	*D*-erythro-isocitric acid	30810-51-6	192.14	-1.34	4	7	65.43	-1.67	0.04
C59	*P. ginseng*	ginsenoside Rg1	22427-39-0	801.14	1.13	10	14	10.04	-2.27	0.28
C60	*P. ginseng*	ginsenoside Rh8	N/A	622.93	2.62	7	9	4.39	-1.24	0.63
C61	*P. ginseng*	ginsenoyne D	139163-36-3	262.43	4.94	1	2	19.32	1.07	0.13
C62	*P. ginseng*	elemicin	487-11-6	208.28	2.79	0	3	21.94	1.41	0.06
C63	*P. ginseng*	ginsenoside Rh9_qt	N/A	474.8	4.16	3	4	9.61	0.29	0.77
C64	*P. ginseng*	argininyl-fructosyl-glucose_qt	N/A	336.4	-2.88	10	11	11.25	-1.74	0.22
C65	*P. ginseng*	ginsenoside Ra3	90985-77-6	1,241.59	-2.43	17	27	7.19	-4.67	0.02
C66	*P. ginseng*	panaxatriol	32791-84-7	476.82	4.29	3	4	15.42	0.52	0.79
C67	*P. ginseng*	*N*-salicylidene-salicylamine	N/A	227.28	2.9	2	3	95.46	1.12	0.11

C68	*P. ginseng*	stearyl acetate	822-23-1	312.6	7.74	0	2	16.27	1.39	0.19
C69	*P. ginseng*	ginsenoside Rg1_qt	N/A	476.82	4.62	4	4	10.2	0.12	0.78
C70	*P. ginseng*	ginsenoside Rf	52286-58-5	801.14	1.13	10	14	17.74	-2.23	0.24
C71	*P. ginseng*	ginsenoside-Rg4_qt	126223-28-7	458.8	5.84	3	3	10.74	0.25	0.81
C72	*P. ginseng*	ginsenoside Rh4	174721-08-5	620.96	3.84	6	8	3.25	-0.8	0.6
C73	*P. ginseng*	ginsenoside-Rg2	52286-74-5	785.14	2.02	9	13	10.09	-1.94	0.26
C74	*P. ginseng*	folinic acid	N/A	473.5	-0.04	8	14	23.6	-1.7	0.74
C75	*P. ginseng*	1-dodecanol	112-53-8	186.38	4.62	1	1	18.5	1.23	0.03
C76	*P. ginseng*	3-O-*β*-D-glucuronopyranosyl gypsogenin_qt	N/A	470.76	5.59	2	4	23.72	0.22	0.75
C77	*P. ginseng*	L-adenosine	58-61-7	267.28	-2.02	5	8	18.06	-1.42	0.18
C78	*P. ginseng*	psuedohypericin	55954-61-5	520.46	3.8	7	9	16.94	-0.2	0.07
C79	*P. ginseng*	[(3S,4R,5R)-5-[[(2R,3S,4S,5R,6S)-6-(2-acetyl-5-methoxyphenoxy)-3,4,5-trihydroxyoxan-2-yl]methoxy]-3,4-dihydroxyoxolan-3-yl]methyl 3,4,5-trihydroxybenzoate	145898-94-8	612.59	-0.79	8	16	5.61	-2.17	0.63
C80	*P. ginseng*	*β*-bisabolene	495-61-4	204.39	5.33	0	0	29.59	1.88	0.06
C81	*P. ginseng*	kaempferol-3-arabofuranoside	5041-67-8	418.38	0.19	6	10	2.73	-1.08	0.65
C82	*P. ginseng*	ginsenoside Rh3	105558-26-7	618.99	5.47	5	7	12.18	-0.22	0.53
C83	*P. ginseng*	hepanal	111-71-7	204.39	4.36	0	0	53.83	1.86	0.1

C84	*P. ginseng*	ginsenoside RS9	N/A	663.05	4.63	5	8	11.27	-0.76	0.46
C85	*P. ginseng*	tetradecane	629-59-4	198.44	6.76	0	0	15.94	1.79	0.04
C86	*P. ginseng*	ginsenoside Rg2_qt	80952-72-3	476.82	4.62	4	4	20.13	0.17	0.78
C87	*P. ginseng*	pandamine	10233-81-5	552.79	3.87	4	9	16.15	0.11	0.79
C88	*P. ginseng*	ginsenoside Rs2	87733-66-2	1,121.47	-0.31	13	23	8.14	-4.03	0.04
C89	*P. ginseng*	(2*R*,3*S*,4*S*,5*S*,6*R*)-6-(hydroxymethyl)oxane-2,3,4,5-tetrol	7322-31-8	180.18	-2.51	5	6	43.04	-1.82	0.04
C90	*P. ginseng*	methyl linoleate	112-63-0	294.53	6.64	0	2	41.93	1.44	0.17
C91	*P. ginseng*	6'-malonylginsenoside Rd1	N/A	1,195.51	-1.03	15	26	6.7	-4.21	0.03
C92	*P. ginseng*	araloside A	7518-22-1	927.21	1.74	10	18	16.96	-3.32	0.06
C93	*P. ginseng*	3-methylheptane	589-81-1	114.26	3.82	0	0	37.1	1.8	0.01
C94	*P. ginseng*	ginsenoside F2_qt	11021-13-9	460.82	5.79	3	3	29.69	0.5	0.77
C95	*P. ginseng*	(3*S*,5*R*,6*S*,8*R*,9*R*,10*R*,12*R*,13*R*,14*R*,17*S*)-17-[(2*R*)-2-hydroxy-6-methylhept-5-en-2-yl]-4,4,8,10,14-pentamethyl-2,3,5,6,7,9,11,12,13,15,16,17-dodecahydro-1H-cyclopenta[a]phenanthrene-3,6,12-triol	1453-93-6	476.82	4.62	4	4	20.13	0.21	0.78
C96	*P. ginseng*	girinimbin	23095-44-5	263.36	4.6	1	1	61.22	1.72	0.31
C97	*P. ginseng*	ditertbutyl phthalate	30448-43-2	278.38	3.4	0	4	43.67	1.13	0.13

C98	*P. ginseng*	ginsenoside Rb1	41753-43-9	1,109.46	-1.2	15	23	6.24	-3.99	0.04
C99	*P. ginseng*	5-isobutylnonane	62185-53-9	184.41	5.89	0	0	6.08	1.82	0.03
C100	*P. ginseng*	panaxynol	21852-80-2	244.41	5.68	1	1	42.44	1.52	0.1
C101	*P. ginseng*	ginsenoside Rg4	126223-28-7	767.12	3.23	8	12	10.9	-1.77	0.27
C102	*P. ginseng*	methyl margarate	1731-92-6	284.54	7.08	0	2	17.41	1.37	0.14
C103	*P. ginseng*	methyl palmitate	112-39-0	270.51	6.62	0	2	18.09	1.37	0.12
C104	*P. ginseng*	malonic acid	141-82-2	104.07	-0.44	2	4	21.69	-0.23	0.01
C105	*P. ginseng*	neohexane	75-83-2	86.2	2.65	0	0	37.81	1.77	0.01
C106	*P. ginseng*	alexandrin	474-58-8	576.95	6.34	4	6	20.63	-0.1	0.63
C107	*P. ginseng*	adenosine triphosphate	N/A	491.22	-4.3	8	16	8.23	-3.1	0.58
C108	*P. ginseng*	ginsenoside Rh8_qt	N/A	460.77	4.37	4	4	14.72	-0.04	0.8
C109	*P. ginseng*	2,3,4-trimethyldecane	62238-15-7	184.41	5.69	0	0	16.15	1.82	0.03
C110	*P. ginseng*	1-hexadecyne	629-74-3	222.46	7.86	0	0	3.94	1.89	0.06
C111	*P. ginseng*	ginsenoside Rh1_qt	63223-86-9	476.82	4.62	4	4	8.91	0.12	0.78
C112	*P. ginseng*	nepetin	520-11-6	316.28	2.05	4	7	26.75	0.37	0.31
C113	*P. ginseng*	ginsenoside Re	52286-59-6	933.27	0.06	12	18	5.42	-2.88	0.13
C114	*P. ginseng*	tridecanoic acid	638-53-9	214.39	5	1	2	22.32	1.04	0.05
C115	*P. ginseng*	ginsenoside La_qt	N/A	458.8	5.33	2	3	15.7	0.65	0.78
C116	*P. ginseng*	campesteryl ferulate	20972-07-0	576.94	9.86	1	4	22.1	1.1	0.59
C117	*P. ginseng*	9-hexadecenoic acid	10030-73-6	254.46	5.92	1	2	35.78	1.1	0.1
C118	*P. ginseng*	ginsenoside Rg3	14197-60-5	785.14	2.3	9	13	13.69	-1.65	0.22
C119	*P. ginseng*	malvic acid	503-05-9	280.5	6.44	1	2	30.99	1.22	0.15

C120	*P. ginseng*	ginsenoside Rg3	14197-60-5	785.14	2.3	9	13	12.43	-1.75	0.22
C121	*P. ginseng*	3,4-dmethylheptane	922-28-1	128.29	4.07	0	0	46.67	1.78	0.01
C122	*P. ginseng*	ginsenoside Ra1	83459-41-0	1,211.56	-1.92	16	26	7.09	-4.51	0.02
C123	*P. ginseng*	malonylginsenoside Rd_qt	N/A	460.82	5.79	3	3	29.69	0.47	0.77
C124	*P. ginseng*	dibutyl benzene-1,2-dicarboxylate	84-74-2	278.38	4.2	0	4	64.54	0.8	0.13
C125	*P. ginseng*	ginsenoside Ra1_qt	68406-26-8	460.82	5.79	3	3	29.69	0.3	0.77
C126	*P. ginseng*	paeonol	552-41-0	166.19	1.29	1	3	28.79	0.93	0.04
C127	*P. ginseng*	methyl (*Z*)-icos-11-enoate	2390-09-2	324.61	8	0	2	29.49	1.41	0.23
C128	*P. ginseng*	pamalic acid	500-37-8	346.36	3.77	3	7	6	0.35	0.3
C129	*P. ginseng*	*α*-D-mannopyranuronic acid	N/A	194.16	-2.31	5	7	56.17	-2.14	0.06
C130	*P. ginseng*	ginsenoside Rb2	11021-13-9	1,079.43	-0.69	14	22	5.98	-4.07	0.04
C131	*P. ginseng*	*α*-guttiferin	N/A	452.59	6.41	3	6	4.43	0.59	0.65
C132	*P. ginseng*	ginsenoside F3	62025-50-7	771.11	1.64	9	13	13.15	-1.75	0.3
C133	*P. ginseng*	ginsenoside Rc	11021-14-0	1,079.43	-0.69	14	22	8.13	-3.86	0.04
C134	*P. ginseng*	ginsenoyne A	139163-34-1	258.39	4.3	1	2	66.22	0.99	0.13
C135	*P. ginseng*	dianthramine	136945-65-8	289.26	2.05	5	7	40.45	-0.23	0.2
C136	*P. ginseng*	2-formylpyrrole	1003-29-8	95.11	0.97	1	1	41.58	1.12	0.01
C137	*P. ginseng*	*β*-sitosterol	83-46-5	414.79	8.08	1	1	36.91	1.32	0.75
C138	*P. ginseng*	(3R,5R,8R,9R,10R,12R,13R,14R,17S)-17-[(2S)-2-hydroxy-6-methylhept-5-en-2-yl]-4,4,8,10,14-pentamethyl-	41753-43-9	460.82	5.79	3	3	29.69	0.35	0.77

		2,3,5,6,7,9,11,12,13,15,16,17-dodecahydro-1H-cyclopenta[a]phenanthrene-3,12-diol								
C139	*P. ginseng*	octane	111-65-9	114.26	4.02	0	0	29.72	1.78	0.01
C140	*P. ginseng*	2,6-dimethyl-3,7-octadiene-2,6-diol	51276-34-7	170.28	1.33	2	2	52.4	0.47	0.03
C141	*P. ginseng*	4-methyldodecane	6117-97-1	184.41	6.1	0	0	6.39	1.81	0.03
C142	*P. ginseng*	pangamic acid	11006-56-7	436.62	1.11	5	10	10.08	-0.92	0.32
C143	*P. ginseng*	ginsenoside Re_qt	52286-59-6	476.82	4.62	4	4	20.13	0.27	0.78
C144	*P. ginseng*	ginsenoside Rs1_qt	68406-26-8	460.82	5.79	3	3	29.69	0.3	0.77
C145	*P. ginseng*	ginsenoyne C	139163-35-2	276.41	3.53	3	3	43.38	0.15	0.13
C146	*P. ginseng*	12-O-nicotinoylisolineolone	N/A	469.63	1.32	3	7	20.7	-0.54	0.83
C147	*P. ginseng*	celabenzine	53938-08-2	379.55	2.29	2	5	101.88	0.77	0.49
C148	*P. ginseng*	malonylginsenoside Rd	N/A	1,033.35	0.71	12	21	8.84	-3.72	0.07
C149	*P. ginseng*	7*α*-*L*-rhamnosyl-6-methoxylutcolin	35682-55-4	462.44	1.03	6	11	15.03	-0.69	0.79
C150	*P. ginseng*	(1*S*,4*E*,8*E*,10*R*)-4,8,11,11-tetramethylbicyclo[8.1.0]undeca-4,8-diene	24703-35-3	204.39	4.7	0	0	21.69	1.86	0.08
C151	*P. ginseng*	methylselenocysteine	26046-90-2	182.1	-0.27	3	3	35.74	0.01	0.01
C152	*P. ginseng*	(2*S*,3*R*,4*S*,5*S*,6*R*)-2-[(2*S*)-2-[(3*S*,5*R*,8*R*,9*R*,10*R*,12*R*,13*R*,14*R*,17*S*)-3-[(2*R*,3*R*,4*S*,5*S*,6*R*)-4,5-	52705-93-8	947.3	0.55	12	18	5.5	-3.17	0.09

		dihydroxy-6-(hydroxymethyl)-3-[(2*S*,3*R*,4*S*,5*S*,6*R*)-3,4,5-trihydroxy-6-(hydroxymethyl)oxan-2-yl]oxyoxan-2-yl]oxy-12-hydroxy-4,4,8,10,14-pentamethyl-2,3,5,6,7,9,11,12,13,15,1								
C153	*P. ginseng*	ginsenoside-Rh4	174721-08-5	620.96	3.84	6	8	5.22	-0.73	0.6
C154	*P. ginseng*	*N*, *N*-dimethyldecanamide	14433-76-2	199.38	3.44	0	2	55.5	1.51	0.04
C155	*P. ginseng*	vulgarin	3162-56-9	264.35	1.31	1	4	29.21	0.03	0.2
C156	*P. ginseng*	neocnidilide	4567-33-3	194.3	3.37	0	2	83.83	1.23	0.07
C157	*P. ginseng*	ginsenoside-Rb2	11021-13-9	1,079.43	-0.69	14	22	6.02	-3.92	0.04
C158	*P. ginseng*	(*R*)-(+)-citronellal	2385-77-5	154.28	3.02	0	1	50.78	1.37	0.02
C159	*P. ginseng*	undecane-3,6-dimethyl	17301-28-9	184.41	5.89	0	0	12.85	1.79	0.03
C160	*P. ginseng*	ginsenoside-Rh1	63223-86-9	638.98	2.87	7	9	3.94	-1.1	0.57
C161	*P. ginseng*	20-hexadecanoylingenol	N/A	586.94	7.38	3	6	28.2	0.3	0.68
C162	*P. ginseng*	ginsenoside Rg3_qt	38243-03-7	460.82	5.86	3	3	14.83	0.49	0.77
C163	*P. ginseng*	cis-widdrol α-epoxide	N/A	238.41	2.6	1	2	69.04	1.07	0.15
C164	*P. ginseng*	ginsenoside F1	53963-43-2	638.98	2.94	7	9	4.05	-1.24	0.61
C165	*P. ginseng*	dauricine	524-17-4	624.84	7.22	1	8	23.65	0.9	0.37
C166	*P. ginseng*	3-[[(2*S*)-2,4-dihydroxy-3,3-dimethylbutanoyl]amino]propanoic acid	79-83-4	219.27	-0.98	4	6	21.29	-0.85	0.06

C167	*P. ginseng*	ginsenoside Rh3_qt	105558-26-7	456.83	7.21	2	2	8.06	1.04	0.78
C168	*P. ginseng*	ginsenoside Rh6	N/A	670.98	1.88	8	11	6.46	-1.67	0.55
C169	*P. ginseng*	ginsenoside Rc	11021-14-0	1,079.43	-0.69	14	22	8.16	-3.97	0.04
C170	*P. ginseng*	7-(*β*-xylosyl)cephalomannine	N/A	962.15	1.97	6	18	27.33	-1.67	0.17
C171	*P. ginseng*	ginsenoside F2	62025-49-4	785.14	2.22	9	13	37.03	-2.04	0.25
C172	*P. ginseng*	ginsenoside Rc_qt	11021-13-9	460.82	5.79	3	3	29.69	0.5	0.77
C173	*P. ginseng*	ginsenoside Rs2_qt	68406-26-8	460.82	5.79	3	3	29.69	0.3	0.77
C174	*P. ginseng*	5-[(3aS,6R,6aR)-2-keto-1,3,3a,4,6,6a-hexahydrothieno[3,4-d]imidazol-6-yl]valeric acid	22879-79-4	244.35	0.65	3	5	75.75	-0.04	0.1
C175	*P. ginseng*	ginsenoside Ro_qt	N/A	455.77	5.74	1	3	17.62	0.38	0.76
C176	*P. ginseng*	ginsenoside Rh5_qt	N/A	474.80	4	4	12.22	-0.08	-0.92	0.26
C177	*P. ginseng*	ginsenoyne B	139035-29-3	294.85	4.74	2	2	39.79	0.79	0.13
C178	*P. ginseng*	(1R,4S,4aR,8aR)-4-isopropyl-1,6-dimethyl-3,4,4a,7,8,8a-hexahydro-2H-naphthalen-1-ol	481-34-5	222.41	3.78	1	1	31.67	1.32	0.09
C179	*P. ginseng*	methyl tricosanoate	2433-97-8	368.72	9.81	0	2	14.61	1.43	0.33
C180	*P. ginseng*	3-O-*β*-D-glucuronopyranosyl gypsogenin	N/A	646.9	4.05	5	10	8.68	-0.78	0.32
C181	*P. ginseng*	(*Z*,*Z*)-*α*-farnesene	502-61-4	204.39	5.46	0	0	8.47	1.92	0.05
C182	*P. ginseng*	ginsenoside Rg5	186763-78-0	767.12	3.31	8	12	6.15	-1.92	0.23
C183	*P. ginseng*	protopanaxadiol	7755-01-3	460.82	5.79	3	3	29.69	0.57	0.77

C184	*P. ginseng*	ginsenoside R0	34367-04-9	957.24	1.07	11	19	2.1	-3.57	0.04
C185	*P. ginseng*	kaempferol	520-18-3	286.25	1.77	4	6	41.88	0.26	0.24
C186	*P. ginseng*	ginsenoside Rg7	N/A	801.14	1.25	10	14	34	-2.46	0.24
C187	*P. ginseng*	ginsenoside Ra2	83459-42-1	1,211.56	-1.92	16	26	7.62	-4.43	0.02
C188	*P. ginseng*	ginsenoside Rh9	N/A	636.96	2.41	6	9	7.13	-0.96	0.37
C189	*P. ginseng*	notoginsenoside R2	80418-25-3	771.11	1.64	9	13	17.74	-2.22	0.28
C190	*P. ginseng*	pancratistatin	96203-70-2	325.3	-1.94	6	9	13.13	-1.17	0.46
C191	*P. ginseng*	methyl myristate	124-10-7	242.45	5.71	0	2	19.68	1.36	0.08
C192	*P. ginseng*	(1*R*,4*E*,7*E*,11*R*)-1,5,9,9-tetramethyl-12-oxabicyclo[9.1.0]dodeca-4,7-diene	19888-33-6	220.39	3.8	0	1	23.66	1.58	0.1
C193	*P. ginseng*	ε-cadinene	N/A	204.39	4.85	0	0	16.41	1.82	0.08
C194	*P. ginseng*	ginsenoside Rg7_qt	114019-97-5	476.82	4.74	4	4	10.73	-0.02	0.79
C195	*P. ginseng*	chrysanthemaxanthin	27780-11-6	584.96	8.24	2	3	38.72	0.51	0.58
C196	*P. ginseng*	darutoside	19716-26-8	574.93	5.89	4	6	21.32	-0.26	0.63
C197	*P. ginseng*	ginsenoside Rh2_qt	N/A	460.82	5.79	3	3	29.69	0.47	0.77
C198	*P. ginseng*	notoginsenoside R6	N/A	963.3	-0.62	13	19	4.7	-3.46	0.12
C199	*P. ginseng*	ginsenoside Rb3_qt	80330-77-4	460.82	5.79	3	3	29.69	0.33	0.77
C200	*P. ginseng*	ginsenoside Rg3_qt	N/A	460.82	5.79	3	3	29.69	0.31	0.77
C201	*P. ginseng*	L-erythro-isocitric acid	30810-51-6	192.14	-1.34	4	7	32.95	-1.46	0.04
C202	*P. ginseng*	(*Z*)-2-methyl-5-[(1S,2R,4R)-2-methyl-3-methylene-2-	77-42-9	220.39	3.66	1	1	35.28	1.28	0.09

		norbornanyl]pent-2-en-1-ol								
C203	*P. ginseng*	2-methyltridecane	1560-96-9	198.44	6.55	0	0	5.75	1.81	0.04
C204	*P. ginseng*	suffruticoside A_qt1	N/A	302.26	-0.93	6	9	13.9	-1.35	0.2
C205	*P. ginseng*	3,5-dimethyl-*p*-anisic acid	21553-46-8	180.22	2.39	1	3	61.11	0.75	0.05
C206	*P. ginseng*	3-methylundecane	1002-43-3	170.38	5.64	0	0	6.57	1.79	0.02
C207	*P. ginseng*	deoxyharringtonine	36804-95-2	515.66	3.13	1	9	39.27	0.19	0.81
C208	*P. ginseng*	methyl stearate	112-61-8	298.57	7.53	0	2	16.8	1.41	0.16
C209	*P. ginseng*	ginsenoside Ra0	N/A	1,271.62	-2.94	18	28	7.3	-5.56	0.01
C210	*P. ginseng*	frutinone A	38210-27-4	264.24	2.7	0	4	65.9	0.89	0.34
C211	*P. ginseng*	ginsenoside Rh2	78214-33-2	622.98	4.04	6	8	36.32	-0.51	0.56
C212	*P. ginseng*	γ-selinene	515-17-3	204.39	4.95	0	0	22.58	1.84	0.08
C213	*P. ginseng*	(3*S*,5*R*,8*R*,9*R*,10*R*,12*R*,13*R*,14*R*,17S)-17-[(2*S*)-2-hydroxy-6-methylhept-5-en-2-yl]-4,4,8,10,14-pentamethyl-2,3,5,6,7,9,11,12,13,15,16,17-dodecahydro-1H-cyclopenta[a]phenanthrene-3,12-diol	N/A	460.82	5.79	3	3	29.69	0.37	0.77
C214	*P. ginseng*	(2*R*,3*S*,4*S*,5*R*,6*R*)-2-(hydroxymethyl)-6-[[(3*S*,5*R*,8*R*,9*R*,10*R*,12*R*,13*R*,14*R*,17*S*)-12-hydroxy-4,4,8,10,14-	105558-26-7	604.96	5.01	5	7	12.09	-0.35	0.59

		pentamethyl-17-[(2Z)-6-methylhepta-2,5-dien-2-yl]-2,3,5,6,7,9,11,12,13,15,16,17-dodecahydro-1H-cyclopenta[a]phenanthren-3-yl]oxy]oxane-3,4,5-triol								
C215	*P. ginseng*	ginsenoside Rd2_qt	68406-26-8	460.82	5.79	3	3	29.69	0.3	0.77
C216	*P. ginseng*	ginsenoside Rg1	22427-39-0	801.14	1.13	10	14	9.03	-2.24	0.28
C217	*P. ginseng*	notoginsenoside Fe_qt	N/A	460.82	5.79	3	3	29.69	0.33	0.77
C218	*P. ginseng*	ginsenoside Rh7	N/A	636.96	2.69	6	9	7.72	-1.17	0.6
C219	*P. ginseng*	diisooctyl phthalate	27554-26-3	390.62	7.44	0	4	43.59	0.79	0.39
C220	*P. ginseng*	*β*-elemene	87-44-5	204.39	4.79	0	0	25.63	1.84	0.06
C221	*P. ginseng*	ginsenoside Ro	34367-04-9	957.24	1.23	11	19	1.98	-2.86	0.05
C222	*P. ginseng*	suchilactone	50816-74-5	368.41	3.73	0	6	57.52	0.82	0.56
C223	*P. ginseng*	ginsenoside F1_qt	53963-43-2	476.82	4.69	4	4	9.67	0.07	0.79
C224	*P. ginseng*	ginsenoside Rh1	63223-86-9	638.98	2.87	7	9	3.86	-1.17	0.57
C225	*P. ginseng*	inermin	19908-48-6	284.28	2.44	1	5	65.83	0.91	0.54
C226	*P. ginseng*	(+)-maalioxide	53625-18-6	222.41	3.47	0	1	55.93	1.59	0.13
C227	*P. ginseng*	6,8-nonacosanediol	96850-33-8	188.35	2.79	2	2	17.79	0.56	0.03
C228	*P. ginseng*	ginsenoside Rb1	41753-43-9	1,109.46	-1.2	15	23	6.24	-3.99	0.04
C229	*P. ginseng*	ginsenoyne E	126146-63-2	258.39	4.93	0	2	36.53	1.05	0.13
C230	*P. ginseng*	2,6,10,15-tetramethylheptadecane	54833-48-6	296.65	9.13	0	0	13.73	1.85	0.13

C231	*P. ginseng*	7-tetradecyne	35216-11-6	194.4	6.44	0	0	20.07	1.93	0.04
C232	*P. ginseng*	acetal	73506-93-1	118.2	0.78	0	2	26.4	1.25	0.01
C233	*P. ginseng*	oleanane	471-67-0	412.82	8.66	0	0	6.69	1.83	0.76
C234	*P. ginseng*	panaxytriol	87005-03-6	278.43	3.92	3	3	33.76	0.06	0.13
C235	*P. ginseng*	gomisin B	58546-55-7	514.62	2.73	1	9	31.99	0.6	0.83
C236	*P. ginseng*	fumarine	130-86-9	353.4	2.95	0	6	59.26	0.56	0.83
C237	*P. ginseng*	ginsenoside Rs1_qt	68406-26-8	460.82	5.79	3	3	29.69	0.3	0.77
C238	*P. ginseng*	loxanol V	112-72-1	214.44	5.53	1	1	14.19	1.27	0.05
C239	*P. ginseng*	ginsenoside Rh7_qt	N/A	474.8	4.44	3	4	29.23	0.08	0.78
C240	*P. ginseng*	trifolirhizin	6807-83-6	446.44	0.54	4	10	7.62	-0.83	0.79
C241	*P. ginseng*	ginsenoside Rd	52705-93-8	963.15	1.06	11	17	5.02	-3.05	0.11
C242	*P. ginseng*	argininyl-fructosyl-glucose	N/A	498.56	-4.63	13	16	0.74	-3.12	0.62
C243	*P. ginseng*	ginsenoside-Ra2_qt	68406-26-8	460.82	5.79	3	3	29.69	0.3	0.77
C244	*P. ginseng*	ginsenoside Rg2	80952-72-3	785.14	2.02	9	13	10.09	-1.96	0.26
C245	*P. ginseng*	ginsenoside F3_qt	62025-50-7	476.82	4.62	4	4	20.13	0.02	0.78
C246	*P. ginseng*	ginsenoside Rb2_qt	11021-13-9	460.82	5.79	3	3	29.69	0.5	0.77
C247	*P. ginseng*	6'-malonylginsenoside Rd1_qt1	N/A	460.82	5.79	3	3	29.69	0.56	0.77
C248	*P. ginseng*	ginsenoside La	123617-34-5	783.12	1.84	8	13	17.74	-1.9	0.14
C249	*P. ginseng*	ginsenoside Rd2	83480-64-2	917.27	1.05	11	17	5.01	-3.05	0.11
C250	*P. ginseng*	δ-elemene	20307-84-0	204.39	4.73	0	0	25.99	1.84	0.06
C251	*P. ginseng*	ginsenoside Rh4_qt	174721-08-5	458.8	5.59	3	3	9.84	0.36	0.78
C252	*P. ginseng*	maltose	133-99-3	342.34	-4.26	8	11	1.8	-2.7	0.24

C253	*P. ginseng*	stigmasterol	83-48-7	412.77	7.64	1	1	43.83	1.44	0.76
C254	*P. ginseng*	pentadecane	629-62-9	212.47	7.22	0	0	13.98	1.81	0.05
C255	*P. ginseng*	arachidonate	506-32-1	304.52	6.41	1	2	45.57	1.27	0.2
C256	*P. ginseng*	ginsenoside Rg5_qt	N/A	442.8	6.8	2	2	39.56	0.88	0.79
C257	*P. ginseng*	ginsenoside Rd_qt	62025-49-4	460.82	5.79	3	3	12.23	0.65	0.77
C258	*P. ginseng*	ginsenoside Ra0_qt	N/A	460.82	5.79	3	3	29.69	0.3	0.77
C259	*P. ginseng*	malonylginsenoside Rc_qt1	N/A	460.82	5.79	3	3	29.69	0.45	0.77
C260	*P. ginseng*	alexandrin_qt	N/A	414.79	8.08	1	1	36.91	1.3	0.75
C261	*P. ginseng*	notoginsenoside R2_qt	N/A	476.82	4.62	4	4	20.13	0.2	0.78
C262	*P. ginseng*	ginsenoside Rh1_qt	N/A	476.82	4.62	4	4	20.13	-0.02	0.78
C263	*P. ginseng*	mycosinol	111768-19-5	214.23	1.46	1	3	82.12	0.87	0.09
C264	*P. ginseng*	ginsenoside Rh4_qt	N/A	458.8	5.59	3	3	31.11	0.5	0.78
C265	*P. ginseng*	ginsenoside Rh6_qt	N/A	508.82	3.63	5	6	10.51	-0.56	0.8
C266	*P. ginseng*	3-ethyl-3-methylheptane	17302-01-1	142.32	4.48	0	0	37.33	1.81	0.02
C267	*P. ginseng*	aposiopolamine	N/A	271.34	1.39	1	4	66.65	0.66	0.22
C268	*P. ginseng*	ginsenoside Rs1	87733-67-3	1,121.47	-0.31	13	23	6.27	-3.69	0.04
C269	*P. ginseng*	ginsenoside Rs1	87733-67-3	1,121.47	-0.31	13	23	6.27	-3.69	0.04
C270	*P. ginseng*	malonylginsenoside Rc	N/A	1,165.48	-0.52	14	25	7.84	-4.27	0.03
C271	*P. ginseng*	linoleic	60-33-3	280.5	6.39	1	2	41.9	1.23	0.14
C272	*P. ginseng*	dammarane	545-22-2	414.84	9.53	0	0	19.73	1.81	0.7
C273	*P. ginseng*	13-tetradecenyl acetate	56221-91-1	254.46	5.52	0	2	36.76	1.36	0.1
C274	*P. ginseng*	ginsenoside Ra3_qt	68406-26-8	460.82	5.79	3	3	29.69	0.3	0.77

C275	*P. ginseng*	ginsenoside Rb3	68406-26-8	1,079.43	-0.69	14	22	7.84	-3.98	0.04
C276	*P. ginseng*	panaxadiol	19666-76-3	460.82	5.46	2	3	33.09	0.82	0.79
C277	*P. ginseng*	malkangunin	52691-06-2	432.56	1.84	2	7	57.71	0.22	0.63
C278	*P. ginseng*	5-methyl-tetradecane	25117-32-2	212.47	7.01	0	0	16.15	1.83	0.05
C279	*P. ginseng*	(4a*S*,6a*R*,6a*S*,6b*R*,8a*R*,10*S*,12a*R*,14b*R*)-10-hydroxy-2,2,6a,6b,9,9,12a-heptamethyl-1,3,4,5,6,6a,7,8,8a,10,11,12,13,14b-tetradecahydropicene-4a-carboxylic acid	6892-79-1	456.78	6.42	2	3	14.36	0.6	0.76
C280	*P. ginseng*	5-heptadec-12-enylresorcinol	N/A	346.61	8.64	2	2	3.29	1.38	0.32
C281	*P. ginseng*	methyl palmitelaidate	10030-74-7	268.49	6.17	0	2	34.61	1.4	0.12
C282	*P. ginseng*	pentadecylic acid	1002-84-2	242.45	5.91	1	2	20.18	1.08	0.08
C283	*P. ginseng*	ginsenoside Rf	52286-58-5	801.14	1.13	10	14	15.33	-2.12	0.24
C284	*P. ginseng*	16-oxoseratenediol	24513-51-7	456.78	5.52	2	3	15.1	0.4	0.75
C285	*P. ginseng*	*α*-cubebol	81-34-5	208.38	3.28	1	1	64.81	1.32	0.09
C286	*P. ginseng*	octanal	124-13-0	128.24	2.77	0	1	19.07	1.3	0.01
C287	*P. ginseng*	2-heptodecanone	2922-51-2	254.51	6.35	0	1	15.31	1.49	0.09
C288	*P. ginseng*	gomisin A	58546-54-6	416.51	3.85	1	7	30.69	0.63	0.78
C289	*P. ginseng*	raffinose	17629-30-0	504.5	-6.06	11	16	11.79	-3.91	0.66
C290	*P. ginseng*	campesterol	474-62-4	400.76	7.63	1	1	37.58	1.34	0.71
C291	*P. ginseng*	heptadekan	629-78-7	240.53	8.13	0	0	8.64	1.84	0.07

C292	*P. ginseng*	eicosane	112-95-8	282.62	9.5	0	0	8.46	1.83	0.13
C293	*P. ginseng*	calarene	17334-55-3	236.39	3.44	1	2	16.81	1	0.14
C294	*P. ginseng*	xylose	6763-34-4	150.15	-2	4	5	58.74	-1.16	0.03
C295	*P. ginseng*	spermine	115-04-8	202.4	-1.5	6	4	26.81	-0.21	0.04
C296	*P. ginseng*	choline	62-49-7	104.2	-1.57	1	1	0.47	0.86	0.01
C297	*P. ginseng*	*δ*-cadinol	91-17-8	204.39	4.94	0	0	17.13	1.87	0.08
C298	*P. ginseng*	girinimbin	23095-44-5	263.36	4.6	1	1	61.22	1.72	0.31
C299	*P. ginseng*	pyrrole-2-Aldehyde	1003-29-8	95.11	0.97	1	1	41.58	1.12	0.01
C300	*P. ginseng*	ramalic acid	500-37-8	346.36	3.77	3	7	6	0.35	0.3
C301	*P. ginseng*	trifolin	23627-87-4	448.41	-0.32	7	11	3.1	-1.17	0.74
C302	*P. ginseng*	rhamnose	4469-18-5	164.18	-1.8	4	5	40.73	-1.3	0.03
C303	*P. ginseng*	apohyoscine	535-26-2	285.37	1.93	0	4	59.68	0.84	0.25
C304	*P. ginseng*	*δ*-guaiene	88-84-6	204.39	5.13	0	0	28.21	1.83	0.07
C305	*P. ginseng*	pentadecanoic acid	1002-84-2	242.45	5.91	1	2	20.18	1.08	0.08
C306	*P. ginseng*	pseudohypericin	55954-61-5	520.46	3.8	7	9	16.94	-0.2	0.07
C307	*P. ginseng*	guanosine	118-00-3	283.28	-2.41	6	9	21.43	-1.21	0.21
C308	*P. ginseng*	notoginsenoside R4	N/A	1,241.59	-2.43	17	27	7.32	-4.91	0.02
C309	*P. ginseng*	notoginsenoside R4_qt	N/A	460.82	5.79	3	3	29.69	0.4	0.78
C310	*P. ginseng*	deoxygomisin A	82467-52-5	400.51	5.07	0	6	20.16	1.01	0.75
C311	*P. ginseng*	*n*-tridecane	629-50-5	184.41	6.3	0	0	17.89	1.78	0.03
C312	*P. ginseng*	dianthoside	20847-13-6	288.28	-2.66	4	8	1.99	-0.97	0.17
C313	*P. ginseng*	humulene	19132-75-3	204.39	5.04	0	0	22.98	1.88	0.06

C314	*P. ginseng*	dibutyl phthalate	84-74-2	278.38	4.2	0	4	64.54	0.8	0.13
C315	*P. ginseng*	bicyclogermacrene	67650-90-2	204.39	4.7	0	0	27.33	1.85	0.08
C316	*P. ginseng*	notoginsenoside R1	80418-24-2	933.27	-0.11	12	18	4.27	-2.57	0.13
C317	*P. ginseng*	spermidine	124-20-9	145.29	-1.17	5	3	45.41	-0.12	0.02
C318	*P. ginseng*	palmitoleicacid	373-49-9	254.46	5.92	1	2	35.78	1.18	0.1
C319	*P. ginseng*	riboflavine	130609-39-1	376.41	0.23	5	10	18.18	-1.52	0.5
C320	*P. ginseng*	daucosterol	474-58-8	576.95	6.34	4	6	20.63	0.03	0.63
C321	*P. ginseng*	menthyl acetate	29066-34-0	198.34	3.16	0	2	22.24	1.23	0.05
C322	*P. ginseng*	putrescine	1071-98-3	88.18	-0.83	4	2	81.23	-0.08	0

MW: molecular weight, CLogP: Calculated octanol-water partition coeff., nHdon: number of donor atoms for H-bonds, nHacc: number of acceptor atoms for H-bonds, OB: oral bioavailability, Caco-2: the calculated intestinal epithelial permeability, DL: drug likeness.

_qt represents the molecule with deglycosylation.

Supplementary Table S2 The information of synthetic drugs used for treatment of ulcerative colitis (UC).

ID	Drug	CAS	MW	CLogP	nHDon	nHAc
D1	Tetomilast	145739-56-6	370.42	4.3	1	6
D2	Rivanicline	15585-43-0	162.23	1.21	1	2
D3	Olsalazine	15722-48-2	302.23	4.39	4	8
D4	Balsalazide	80573-04-2	357.32	3.17	4	8
D5	Ozanimod	1306760-87-1	404.47	3.96	2	6
D6	Clindamycin	18323-44-9	424.98	1.09	4	7
D7	ORE-1001	305335-31-3	428.31	-0.9	3	6
D8	Elubrixin	688763-64-6	463.31	1.64	4	5
D9	Sulfasalazine	599-79-1	398.39	3.94	3	8
D10	Budesonide	51333-22-3	430.53	2.73	2	6
D11	Prednisolone	50-24-8	360.44	1.27	3	5
D12	Azathioprine	446-86-6	277.26	1.17	1	6
D13	Mesalazine	89-57-6	153.14	-0.29	3	4
D14	Metronidazole	443-48-1	171.15	-0.46	1	4
D15	Prednisone	53-03-2	358.48	1.66	2	5
D16	Tofacitinib	477600-75-2	312.37	1.24	1	5
D17	Hydrocortisone	50-23-7	362.46	1.28	3	5
D18	Cortisone acetate	50-04-4	402.48	2.1	1	5
D19	Dexamethasone	50-02-2	392.46	1.68	3	5
D20	Methylprednisolone	83-43-2	374.47	1.56	3	5
D21	Mercaptopurine	50-44-2	152.18	-0.12	2	3
D22	Triamcinolone	124-94-7	394.43	0.24	4	6

Supplementary Table S3 Active ingredients and ADME parameters of Renshen and Baizhu.

No.	Compound name	Structure	OB (%)	DL	Herb
M1	(24*S*)-24-propylcholesta-5-ene-3*β*-ol	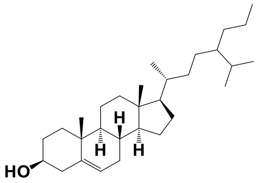	36.23	0.78	*A. macrocephala*
M2	3*β*-acetoxyatractylone	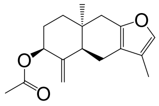	54.07	0.22	*A. macrocephala*
M3	8*β*-ethoxy atractylenolide III	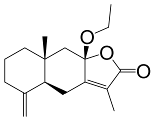	35.95	0.21	*A. macrocephala*
M4#	atractylenolide I	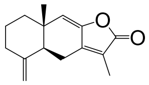	37.37	0.15	*A. macrocephala*
M5#	atractylenolide II	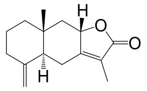	47.5	0.15	*A. macrocephala*
M6	atractylenolide III	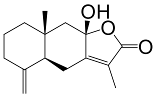	68.11	0.17	*A. macrocephala*
M7#	atractylone	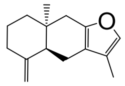	41.1	0.13	*A. macrocephala*
M8	*α*-amyrin	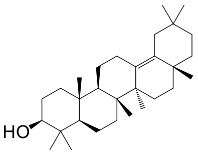	39.51	0.76	*A. macrocephala*
M9#	*β*-eudesmol	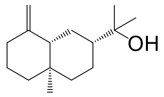	26.09	0.10	*A. macrocephala*

M10#	*β*-caryophyllene	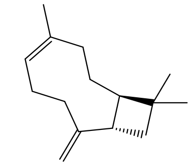	29.7	0.09	*A. macrocephala & P. ginseng*
M11#	araloside A	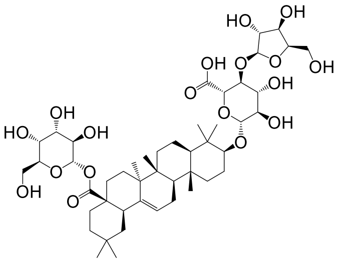	16.96	0.06	*P. ginseng*
M12	celabenzine	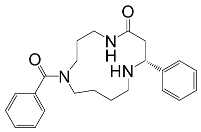	101.88	0.49	*P. ginseng*
M13	chrysanthemaxanthin	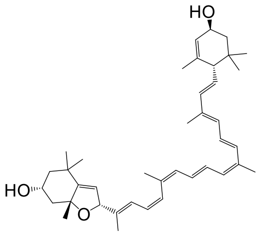	38.72	0.58	*P. ginseng*
M14#	dauricine	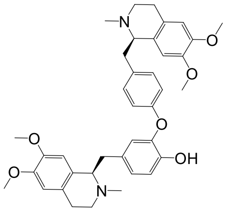	23.65	0.37	*P. ginseng*
M15#	argininyl-fructosyl-glucose	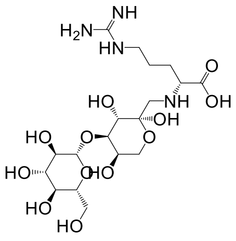	0.74	0.62	*P. ginseng*
M16	dianthramine	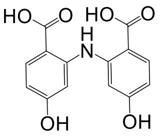	40.45	0.2	*P. ginseng*

M17#	diisooctyl phthalate	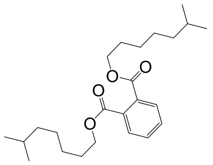	6.24	0.04	*P. ginseng*
M18#	folinic acid	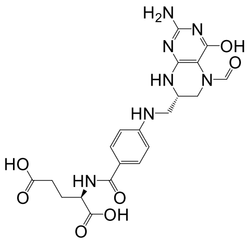	23.6	0.74	*P. ginseng*
M19	frutinone A	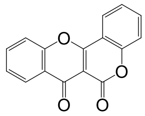	65.9	0.34	*P. ginseng*
M20	fumarine	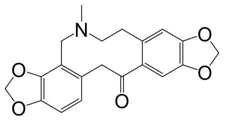	59.26	0.83	*P. ginseng*
M21#	ginsenoside Rb1	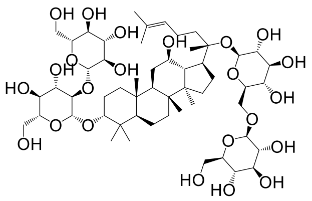	6.24	0.04	*P. ginseng*
M22#	ginsenoside Rb2	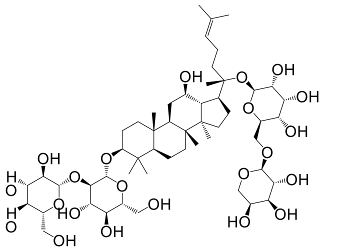	5.98	0.04	*P. ginseng*
M23#	ginsenoside Rb3	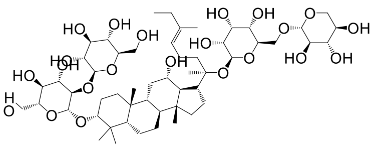	29.69	0.77	*P. ginseng*

M24[#]	ginsenoside Rc	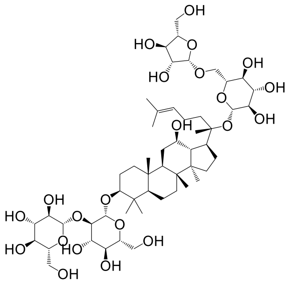	8.13	0.04	*P. ginseng*
M25[#]	ginsenoside Rd	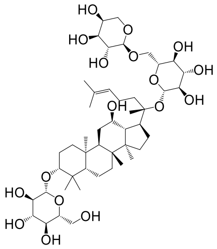	5.02	0.11	*P. ginseng*
M26[#]	ginsenoside Re	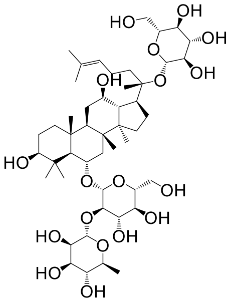	4.27	0.12	*P. ginseng*
M27[#]	ginsenoside Rf	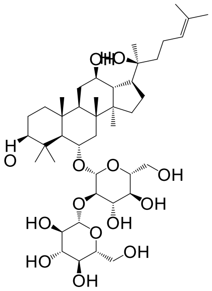	17.74	0.24	*P. ginseng*
M28[#]	ginsenoside Rg1	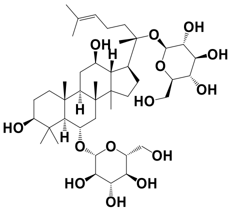	11.21	0.23	*P. ginseng*

M29#	ginsenoside Rg2	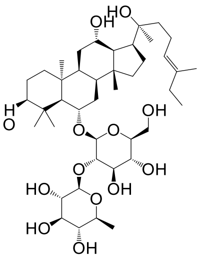	10.09	0.26	*P. ginseng*
M30#	ginsenoside Rg3	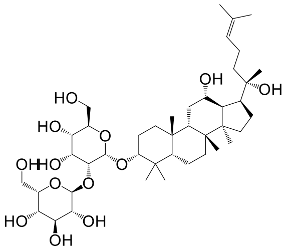	12.43	0.22	*P. ginseng*
M31#	ginsenoside Rg4	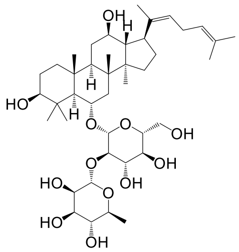	11.34	0.21	*P. ginseng*
M32#	ginsenoside Rg5	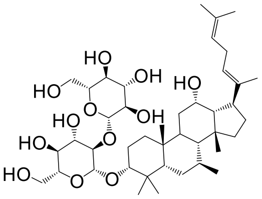	6.15	0.23	*P. ginseng*
M33#	ginsenoside Rg6	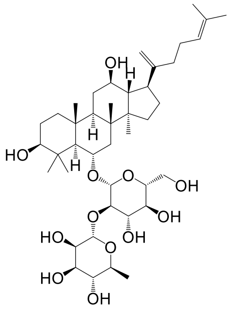	7.03	0.19	*P. ginseng*

M34	ginsenoside Rg7	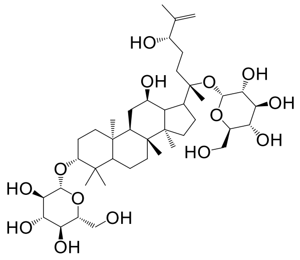	34	0.24	*P. ginseng*
M35	methyl linoleate	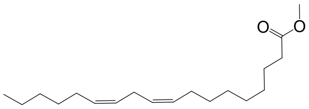	41.93	0.17	*P. ginseng*
M36#	ginsenoside Rh1	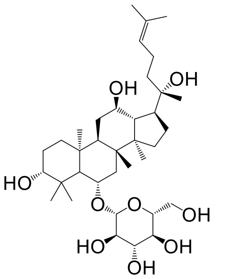	3.86	0.57	*P. ginseng*
M37#	ginsenoside Rh2	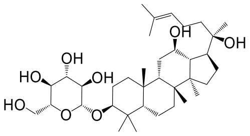	6.54	0.56	*P. ginseng*
M38#	ginsenoside Rh3	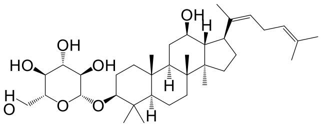	6.18	0.53	*P. ginseng*
M39#	ginsenoside Rh4	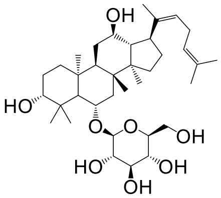	3.25	0.60	*P. ginseng*
M40#	ginsenoside Rh5	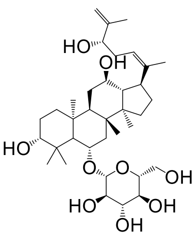	3.23	0.57	*P. ginseng*

M41#	ginsenoside Rh6	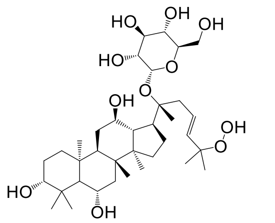	6.46	0.55	*P. ginseng*
M42#	ginsenoside Rh7	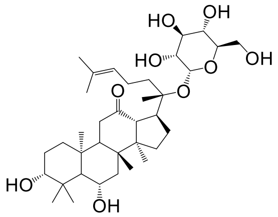	7.72	0.6	*P. ginseng*
M43#	ginsenoside Rh9	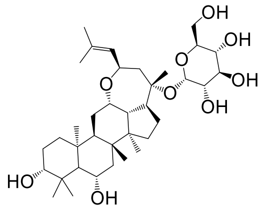	7.13	0.37	*P. ginseng*
M44#	trifolin	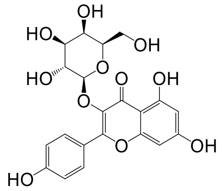	3.10	0.74	*P. ginseng*
M45#	alexandrin	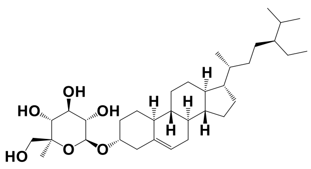	20.46	0.65	*P. ginseng*
M46#	ginsenoside Ro	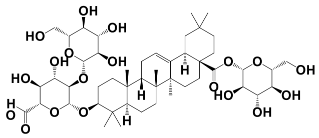	1.98	0.05	*P. ginseng*
M47#	deoxygomisin A	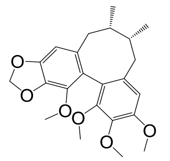	20.16	0.75	*P. ginseng*

M48	girinimbin	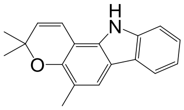	61.22	0.31	*P. ginseng*
M49	gomisin B	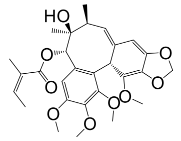	31.99	0.83	*P. ginseng*
M50	inermin	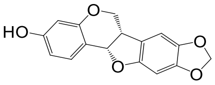	65.83	0.54	*P. ginseng*
M51	kaempferol	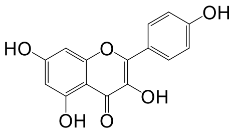	41.88	0.24	*P. ginseng*
M52	malkangunin	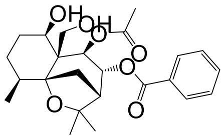	57.71	0.63	*P. ginseng*
M53#	malonylginsenoside Rd	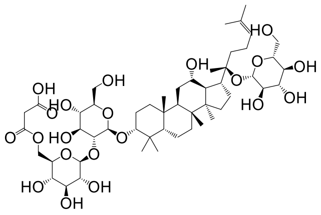	8.84	0.07	*P. ginseng*
M54#	notoginsenoside R2	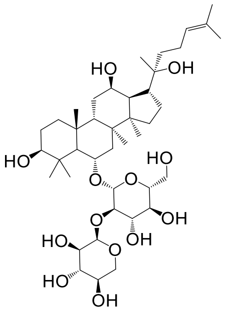	17.74	0.28	*P. ginseng*
M55	panaxadiol	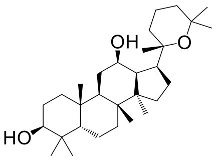	33.09	0.79	*P. ginseng*

M56#	protopanaxadiol	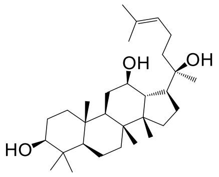	29.69	0.77	*P. ginseng*
M57	stigmasterol	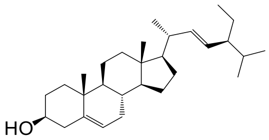	43.83	0.76	*P. ginseng*
M58	suchilactone	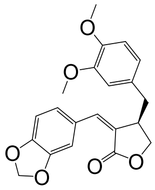	57.52	0.56	*P. ginseng*
M59#	*β*-elemene	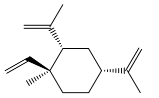	25.63	0.06	*P. ginseng*
M60	*β*-sitosterol	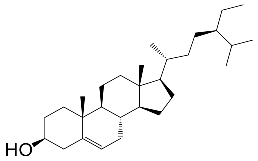	36.91	0.75	*P. ginseng*
M61#	elemicin	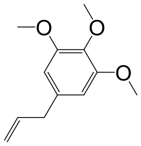	21.94	0.06	*P. ginseng*
M62#	nepetin	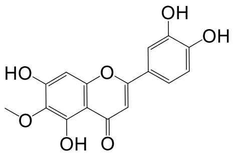	26.75	0.31	*P. ginseng*
M63#	panaxatriol	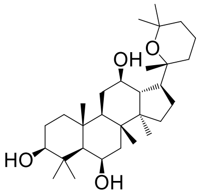	15.42	0.79	*P. ginseng*

M64[#]	campesteryl ferulate	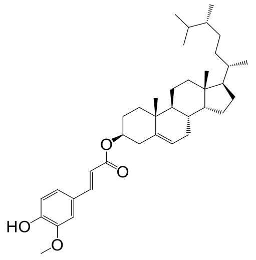	22.10	0.59	*P. ginseng*
M65[#]	paeonol	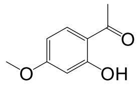	28.79	0.04	*P. ginseng*
M66[#]	*α*-guttiferin	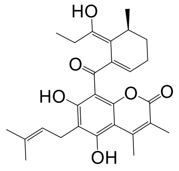	4.43	0.65	*P. ginseng*
M67[#]	trifolirhizin	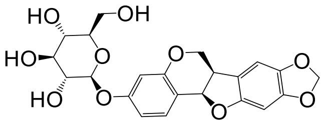	7.62	0.79	*P. ginseng*
M68[#]	kaempferol-3-arabofuranoside	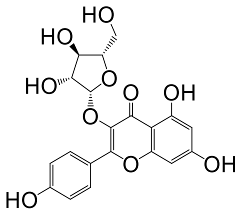	2.73	0.65	*P. ginseng*
M69	apohyoscine	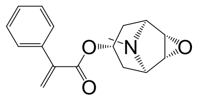	46.47	0.25	*P. ginseng*
M70[#]	guaiene	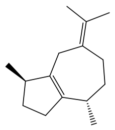	28.21	0.07	*P. ginseng*
M71[#]	*γ*-selinene	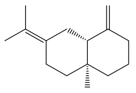	18.02	0.08	*P. ginseng*
M72[#]	dibutyl phthalate	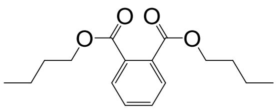	64.54	0.13	*P. ginseng*

M73#	riboflavine	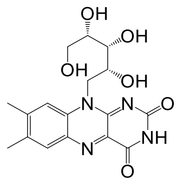	18.18	0.5	*P. ginseng*

OB, oral bioavailability; DL, druglikeness; *A. macrocephala* (*Atractylodis macrocephalae rhizoma*, Baizhu); *P. Ginseng* (*Ginseng radix et rhizoma*, Renshen).

#Compounds with OB < 30% and/or DL < 0.18, yet pharmaceutically validated.

Supplementary Table S4 Target information of Shenzhu Capsule.

No.	Target	Gene name	Origin
T1	ST3GAL1	ST3 beta-galactoside alpha-2,3-sialyltransferase 1	*Homo sapiens*
T2	NOS1	nitric oxide synthase 1	*Homo sapiens*
T3	FAS	Fas cell surface death receptor	*Homo sapiens*
T4	NQO1	NAD(P)H quinone dehydrogenase 1	*Homo sapiens*
T5	STAT1	signal transducer and activator of transcription 1	*Homo sapiens*
T6	GLI1	GLI family zinc finger 1	*Homo sapiens*
T7	PDGFB	platelet derived growth factor subunit B	*Homo sapiens*
T8	LAP3	leucine aminopeptidase 3	*Homo sapiens*
T9	AHR	aryl hydrocarbon receptor	*Homo sapiens*
T10	CYP27B1	cytochrome P450 family 27 subfamily B member 1	*Homo sapiens*
T11	TRPA1	transient receptor potential cation channel subfamily A member 1	*Homo sapiens*
T12	ADRA1D	adrenoceptor alpha 1D	*Homo sapiens*
T13	SNCA	synuclein alpha	*Homo sapiens*
T14	KL	klotho	*Homo sapiens*
T15	CYP17A1	cytochrome P450 family 17 subfamily A member 1	*Homo sapiens*
T16	PLA2G2A	phospholipase A2 group IIA	*Homo sapiens*
T17	APOE	apolipoprotein E	*Homo sapiens*
T18	CYP3A4	cytochrome P450 family 3 subfamily A member 4	*Homo sapiens*
T19	ODC1	ornithine decarboxylase 1	*Homo sapiens*
T20	BAX	BCL2 associated X, apoptosis regulator	*Homo sapiens*
T21	MC4R	melanocortin 4 receptor	*Homo sapiens*
T22	ACADS	acyl-CoA dehydrogenase, C-2 to C-3 short chain	*Homo sapiens*
T23	PPARD	peroxisome proliferator activated receptor delta	*Homo sapiens*
T24	CYP2E1	cytochrome P450 family 2 subfamily E member 1	*Homo sapiens*
T25	RXRA	retinoid X receptor alpha	*Homo sapiens*
T26	LEP	leptin	*Homo sapiens*
T27	DNMT1	DNA methyltransferase 1	*Homo sapiens*
T28	SELP	selectin P	*Homo sapiens*

T29	LGALS9	galectin 9	*Homo sapiens*
T30	MPO	myeloperoxidase	*Homo sapiens*
T31	CCNB1	cyclin B1	*Homo sapiens*
T32	SNAI2	snail family transcriptional repressor 2	*Homo sapiens*
T33	PTEN	phosphatase and tensin homolog	*Homo sapiens*
T34	PPARA	peroxisome proliferator activated receptor alpha	*Homo sapiens*
T35	GAS6	growth arrest specific 6	*Homo sapiens*
T36	CREB1	cAMP responsive element binding protein 1	*Homo sapiens*
T37	FADD	Fas associated via death domain	*Homo sapiens*
T38	AIFM1	apoptosis inducing factor, mitochondria associated 1	*Homo sapiens*
T39	ACOX1	acyl-CoA oxidase 1	*Homo sapiens*
T40	PTGS2	prostaglandin-endoperoxide synthase 2	*Homo sapiens*
T41	FGF2	fibroblast growth factor 2	*Homo sapiens*
T42	IKBKB	inhibitor of kappa light polypeptide gene enhancer in B-cells, kinase beta	*Homo sapiens*
T43	NR3C1	nuclear receptor subfamily 3 group C member 1	*Homo sapiens*
T44	SELE	selectin E	*Homo sapiens*
T45	INS	insulin	*Homo sapiens*
T46	HSP90AB1	heat shock protein 90 alpha family class B member 1	*Homo sapiens*
T47	SPARC	secreted protein acidic and cysteine rich	*Homo sapiens*
T48	CYP1A2	cytochrome P450 family 1 subfamily A member 2	*Homo sapiens*
T49	ANXA1	annexin A1	*Homo sapiens*
T50	EDNRB	endothelin receptor type B	*Homo sapiens*
T51	SERPINE1	serpin family E member 1	*Homo sapiens*
T52	FAAH	fatty acid amide hydrolase	*Homo sapiens*
T53	DRD2	dopamine receptor D2	*Homo sapiens*
T54	CBR1	carbonyl reductase 1	*Homo sapiens*
T55	GSR	glutathione-disulfide reductase	*Homo sapiens*
T56	CAT	catalase	*Homo sapiens*
T57	ITGAL	integrin subunit alpha L	*Homo sapiens*
T58	MAPK14	mitogen-activated protein kinase 14	*Homo sapiens*

T59	TYMS	thymidylate synthetase	*Homo sapiens*
T60	CHRM5	cholinergic receptor muscarinic 5	*Homo sapiens*
T61	TLR2	toll like receptor 2	*Homo sapiens*
T62	CALB1	calbindin 1	*Homo sapiens*
T63	POR	cytochrome p450 oxidoreductase	*Homo sapiens*
T64	TNFSF11	tumor necrosis factor superfamily member 11	*Homo sapiens*
T65	HIF1A	hypoxia inducible factor 1 alpha subunit	*Homo sapiens*
T66	INSR	insulin receptor	*Homo sapiens*
T67	ABCC4	ATP binding cassette subfamily C member 4	*Homo sapiens*
T68	TNF	tumor necrosis factor	*Homo sapiens*
T69	ALDH1A1	aldehyde dehydrogenase 1 family member A1	*Homo sapiens*
T70	RORA	RAR related orphan receptor A	*Homo sapiens*
T71	IGF1	insulin like growth factor 1	*Homo sapiens*
T72	NOX4	NADPH oxidase 4	*Homo sapiens*
T73	IKBKG	inhibitor of kappa light polypeptide gene enhancer in B-cells, kinase gamma	*Homo sapiens*
T74	AGT	angiotensinogen	*Homo sapiens*
T75	XBP1	X-box binding protein 1	*Homo sapiens*
T76	PPARG	peroxisome proliferator activated receptor gamma	*Homo sapiens*
T77	ANPEP	alanyl aminopeptidase, membrane	*Homo sapiens*
T78	SELL	selectin L	*Homo sapiens*
T79	TGFBR1	transforming growth factor beta receptor 1	*Homo sapiens*
T80	CA2	carbonic anhydrase 2	*Homo sapiens*
T81	GPBAR1	G protein-coupled bile acid receptor 1	*Homo sapiens*
T82	HRAS	HRas proto-oncogene, GTPase	*Homo sapiens*
T83	IL13	interleukin 13	*Homo sapiens*
T84	TUBB3	tubulin beta 3 class III	*Homo sapiens*
T85	PTPN2	protein tyrosine phosphatase, non-receptor type 2	*Homo sapiens*
T86	NAMPT	nicotinamide phosphoribosyltransferase	*Homo sapiens*
T87	IFNG	interferon gamma	*Homo sapiens*
T88	IL2	interleukin 2	*Homo sapiens*

T89	NR1H4	nuclear receptor subfamily 1 group H member 4	*Homo sapiens*
T90	TOP1	topoisomerase	*Homo sapiens*
T91	IL6	interleukin 6	*Homo sapiens*
T92	PROC	protein C, inactivator of coagulation factors Va and VIIIa	*Homo sapiens*
T93	VEGFA	vascular endothelial growth factor A	*Homo sapiens*
T94	PDCD4	programmed cell death 4 (neoplastic transformation inhibitor)	*Homo sapiens*
T95	ACE	angiotensin I converting enzyme	*Homo sapiens*
T96	MAP2K1	mitogen-activated protein kinase kinase 1	*Homo sapiens*
T97	CRP	C-reactive protein	*Homo sapiens*
T98	ALOX5	arachidonate 5-lipoxygenase	*Homo sapiens*
T99	HPGD	hydroxyprostaglandin dehydrogenase 15-	*Homo sapiens*
T100	SLCO1B1	solute carrier organic anion transporter family member 1B1	*Homo sapiens*
T101	FBP1	fructose-bisphosphatase 1	*Homo sapiens*
T102	GSTP1	glutathione S-transferase pi 1	*Homo sapiens*
T103	PTGS1	prostaglandin-endoperoxide synthase 1	*Homo sapiens*
T104	PRTN3	proteinase 3	*Homo sapiens*
T105	F2	coagulation factor II, thrombin	*Homo sapiens*
T106	ALPI	alkaline phosphatase, intestinal	*Homo sapiens*
T107	CDK5	cyclin dependent kinase 5	*Homo sapiens*
T108	CXCL8	C-X-C motif chemokine ligand 8	*Homo sapiens*
T109	CACNA1C	calcium voltage-gated channel subunit alpha1 C	*Homo sapiens*
T110	SREBF2	sterol regulatory element binding transcription factor 2	*Homo sapiens*
T111	TLR4	toll like receptor 4	*Homo sapiens*
T112	BGLAP	bone gamma-carboxyglutamate protein	*Homo sapiens*
T113	PIK3CG	phosphatidylinositol-4,5-bisphosphate 3-kinase catalytic subunit gamma	*Homo sapiens*
T114	S100A9	S100 calcium binding protein A9	*Homo sapiens*
T115	TOP2A	topoisomerase (DNA) II alpha	*Homo sapiens*

T116	IGF1R	insulin like growth factor 1 receptor	*Homo sapiens*
T117	XDH	xanthine dehydrogenase	*Homo sapiens*
T118	PRMT1	protein arginine methyltransferase 1	*Homo sapiens*
T119	HSP90AA1	heat shock protein 90 alpha family class A member 1	*Homo sapiens*
T120	HMGCR	3-hydroxy-3-methylglutaryl-CoA reductase	*Homo sapiens*
T121	TERT	telomerase reverse transcriptase	*Homo sapiens*
T122	CASP3	caspase 3	*Homo sapiens*
T123	COL1A1	collagen type I alpha 1 chain	*Homo sapiens*
T124	NFE2L2	nuclear factor, erythroid 2 like 2	*Homo sapiens*
T125	SRC	SRC proto-oncogene, non-receptor tyrosine kinase	*Homo sapiens*
T126	PLAU	plasminogen activator, urokinase	*Homo sapiens*
T127	NR0B1	nuclear receptor subfamily 0 group B member 1	*Homo sapiens*
T128	EDN1	endothelin 1	*Homo sapiens*
T129	ASNS	asparagine synthetase (glutamine-hydrolyzing)	*Homo sapiens*
T130	NR1H3	nuclear receptor subfamily 1 group H member 3	*Homo sapiens*
T131	NPPA	natriuretic peptide A	*Homo sapiens*
T132	Nos2	nitric oxide synthase 2	*Homo sapiens*
T133	PRKAB1	protein kinase AMP-activated non-catalytic subunit beta 1	*Homo sapiens*
T134	VDR	vitamin D (1,25- dihydroxyvitamin D3) receptor	*Homo sapiens*
T135	ABCC1	ATP binding cassette subfamily C member 1	*Homo sapiens*
T136	MMP3	matrix metallopeptidase 3	*Homo sapiens*
T137	JUN	Jun proto-oncogene, AP-1 transcription factor subunit	*Homo sapiens*
T138	IL1B	interleukin 1 beta	*Homo sapiens*
T139	LCT	lactase	*Homo sapiens*
T140	PADI4	peptidyl arginine deiminase 4	*Homo sapiens*
T141	TPMT	thiopurine S-methyltransferase	*Homo sapiens*
T142	ADRB2	adrenoceptor beta 2	*Homo sapiens*
T143	MMP1	matrix metallopeptidase 1	*Homo sapiens*
T144	SLC25A20	solute carrier family 25 member 20	*Homo sapiens*
T145	BCL2	BCL2, apoptosis regulator	*Homo sapiens*

T146	CYP19A1	cytochrome P450 family 19 subfamily A member 1	*Homo sapiens*
T147	NOS3	nitric oxide synthase 3	*Homo sapiens*
T148	MMP9	matrix metallopeptidase 9	*Homo sapiens*
T149	CDKN1A	cyclin dependent kinase inhibitor 1A	*Homo sapiens*
T150	AREG	amphiregulin	*Homo sapiens*
T151	RBP1	retinol binding protein 1	*Homo sapiens*
T152	KDR	kinase insert domain receptor	*Homo sapiens*
T153	ABCG1	ATP binding cassette subfamily G member 1	*Homo sapiens*
T154	CNR1	cannabinoid receptor 1	*Homo sapiens*
T155	F3	coagulation factor III, tissue factor	*Homo sapiens*
T156	SLC10A1	solute carrier family 10 member 1	*Homo sapiens*
T157	ATP4A	ATPase H+/K+ transporting alpha subunit	*Homo sapiens*
T158	AGRN	agrin	*Homo sapiens*
T159	RELA	RELA proto-oncogene, NF-kB subunit	*Homo sapiens*
T160	APP	amyloid beta precursor protein	*Homo sapiens*
T161	ESR2	estrogen receptor 2	*Homo sapiens*
T162	MAPK9	mitogen-activated protein kinase 9	*Homo sapiens*
T163	MAPK1	mitogen-activated protein kinase 1	*Homo sapiens*
T164	TGFB1	transforming growth factor beta 1	*Homo sapiens*
T165	ITGAV	integrin subunit alpha V	*Homo sapiens*
T166	ABCB11	ATP binding cassette subfamily B member 11	*Homo sapiens*
T167	TRPV1	transient receptor potential cation channel subfamily V member 1	*Homo sapiens*
T168	CDK2	cyclin dependent kinase 2	*Homo sapiens*
T169	TNFRSF1A	TNF receptor superfamily member 1A	*Homo sapiens*
T170	AHSA1	activator of Hsp90 ATPase activity 1	*Homo sapiens*
T171	PIK3CA	phosphatidylinositol-4,5-bisphosphate 3-kinase catalytic subunit alpha	*Homo sapiens*
T172	JAK3	Janus kinase 3	*Homo sapiens*
T173	ACHE	acetylcholinesterase (Cartwright blood group)	*Homo sapiens*
T174	NFKB1	nuclear factor kappa B subunit 1	*Homo sapiens*

T175	PTGIS	prostaglandin I2 synthase	*Homo sapiens*
T176	ABCG2	ATP binding cassette subfamily G member 2 (Junior blood group)	*Homo sapiens*
T177	ABCA1	ATP binding cassette subfamily A member 1	*Homo sapiens*
T178	TXNRD1	thioredoxin reductase 1	*Homo sapiens*
T179	SLC19A1	solute carrier family 19 member 1	*Homo sapiens*
T180	RB1	RB transcriptional corepressor 1	*Homo sapiens*
T181	CDK6	cyclin dependent kinase 6	*Homo sapiens*
T182	MAPK8	mitogen-activated protein kinase 8	*Homo sapiens*
T183	S100A8	S100 calcium binding protein A8	*Homo sapiens*
T184	MAOA	monoamine oxidase A	*Homo sapiens*
T185	MTNR1B	melatonin receptor 1B	*Homo sapiens*
T186	LYZ	lysozyme	*Homo sapiens*
T187	AR	androgen receptor	*Homo sapiens*
T188	G6PD	glucose-6-phosphate dehydrogenase	*Homo sapiens*
T189	SI	sucrase-isomaltase	*Homo sapiens*
T190	ABCC2	ATP binding cassette subfamily C member 2	*Homo sapiens*
T191	ACAD8	acyl-CoA dehydrogenase family member 8	*Homo sapiens*
T192	AKT1	AKT serine/threonine kinase 1	*Homo sapiens*
T193	VCAM1	vascular cell adhesion molecule 1	*Homo sapiens*
T194	ABCB1	ATP binding cassette subfamily B member 1	*Homo sapiens*
T195	MGMT	O-6-methylguanine-DNA methyltransferase	*Homo sapiens*
T196	DNAJB9	DnaJ heat shock protein family (Hsp40) member B9	*Homo sapiens*
T197	ADA	adenosine deaminase	*Homo sapiens*
T198	CYP1A1	cytochrome P450 family 1 subfamily A member 1	*Homo sapiens*
T199	ICAM1	intercellular adhesion molecule 1	*Homo sapiens*
T200	TP53	tumor protein p53	*Homo sapiens*
T201	PROS1	protein S	*Homo sapiens*
T202	CYP1B1	cytochrome P450 family 1 subfamily B member 1	*Homo sapiens*
T203	TTR	transthyretin	*Homo sapiens*
T204	ADIPOQ	adiponectin, C1Q and collagen domain containing	*Homo sapiens*

T205	PRKCA	protein kinase C alpha	*Homo sapiens*
T206	GSK3B	glycogen synthase kinase 3 beta	*Homo sapiens*
T207	RNASE1	ribonuclease A family member 1, pancreatic	*Homo sapiens*
T208	HMOX1	heme oxygenase 1	*Homo sapiens*
T209	CHUK	conserved helix-loop-helix ubiquitous kinase	*Homo sapiens*
T210	CNR2	cannabinoid receptor 2	Homo sapiens

Supplementary Table S5 Target information of synthetic drugs used for treatment of ulcerative colitis (UC).

No.	Target	Gene name	Origin
T1	IKBKB	inhibitor of kappa light polypeptide gene enhancer in B-cells, kinase beta	*Homo sapiens*
T2	MMP12	matrix metallopeptidase 12	*Homo sapiens*
T3	ATP4A	ATPase H+/K+ transporting alpha subunit	*Homo sapiens*
T4	IL1A	interleukin 1 alpha	*Homo sapiens*
T5	MC3R	melanocortin 3 receptor	*Homo sapiens*
T6	S100P	S100 calcium binding protein P	*Homo sapiens*
T7	MPO	myeloperoxidase	*Homo sapiens*
T8	MC4R	melanocortin 4 receptor	*Homo sapiens*
T9	IL13RA2	interleukin 13 receptor subunit alpha 2	*Homo sapiens*
T10	SKIV2L2	Ski2 like RNA helicase 2	*Homo sapiens*
T11	ALOX5	arachidonate 5-lipoxygenase	*Homo sapiens*
T12	CCKBR	cholecystokinin B receptor	*Homo sapiens*
T13	PTGS2	prostaglandin-endoperoxide synthase 2	*Homo sapiens*
T14	MADCAM1	mucosal vascular addressin cell adhesion molecule 1	*Homo sapiens*
T15	PLCG2	phospholipase C gamma 2	*Homo sapiens*
T16	HTR3A	5-hydroxytryptamine receptor 3A	*Homo sapiens*
T17	ATP4B	ATPase H+/K+ transporting beta subunit	*Homo sapiens*
T18	PTGS1	prostaglandin-endoperoxide synthase 1	*Homo sapiens*
T19	PTGER4	prostaglandin E receptor 4	*Homo sapiens*
T20	CHRM5	cholinergic receptor muscarinic 5	*Homo sapiens*
T21	IFNG	interferon gamma	*Homo sapiens*
T22	CIITA	class II major histocompatibility complex transactivator	*Homo sapiens*
T23	RFX5	regulatory factor X5	*Homo sapiens*
T24	IL13	interleukin 13	*Homo sapiens*
T25	MIF	macrophage migration inhibitory factor (glycosylation-inhibiting factor)	*Homo sapiens*

T26	RFXAP	regulatory factor X associated protein	*Homo sapiens*
T27	NR3C1	nuclear receptor subfamily 3 group C member 1	*Homo sapiens*
T28	HPS1	HPS1, biogenesis of lysosomal organelles complex 3 subunit 1	*Homo sapiens*
T29	ITGA4	integrin subunit alpha 4	*Homo sapiens*
T30	TNF	tumor necrosis factor	*Homo sapiens*
T31	IL6	interleukin 6	*Homo sapiens*
T32	IL1B	interleukin 1 beta	*Homo sapiens*
T33	SLC7A11	solute carrier family 7 member 11	*Homo sapiens*
T34	PPARG	peroxisome proliferator activated receptor gamma	*Homo sapiens*
T35	HRH2	histamine receptor H2	*Homo sapiens*
T36	ITGB7	integrin subunit beta 7	*Homo sapiens*
T37	LRBA	LPS responsive beige-like anchor protein	*Homo sapiens*
T38	NFKB2	nuclear factor kappa B subunit 2	*Homo sapiens*
T39	ACAT1	acetyl-CoA acetyltransferase 1	*Homo sapiens*
T40	MTOR	mechanistic target of rapamycin	*Homo sapiens*
T41	RPS6KB1	ribosomal protein S6 kinase B1	*Homo sapiens*
T42	ICAM1	intercellular adhesion molecule 1	*Homo sapiens*
T43	KCNMA1	potassium calcium-activated channel subfamily M alpha 1	*Homo sapiens*
T44	MMP9	matrix metallopeptidase 9	*Homo sapiens*
T45	CHUK	conserved helix-loop-helix ubiquitous kinase	*Homo sapiens*
T46	RFXANK	regulatory factor X associated ankyrin containing protein	*Homo sapiens*
T47	NOD2	nucleotide binding oligomerization domain containing 2	*Homo sapiens*
T48	TPMT	thiopurine S-methyltransferase	*Homo sapiens*
T49	NCF4	neutrophil cytosolic factor 4	*Homo sapiens*
T50	FDPS	farnesyl diphosphate synthase	*Homo sapiens*
T51	NPR2	natriuretic peptide receptor 2	*Homo sapiens*
T52	CXCL10	C-X-C motif chemokine ligand 10	*Homo sapiens*

T53	GUCY2C	guanylate cyclase 2C	*Homo sapiens*
T54	SERPINC1	serpin family C member 1	*Homo sapiens*

Supplementary Table S6 Non-disease associated pathways for Shenzhu Capsule.

Term	Count	*P*-Value	Genes
TNF signaling pathway	29	4.64E-21	TNF, PTGS2, MMP9, EDN1, NFKB1, MMP3, VCAM1, AKT1, TNFRSF1A, CASP3, PIK3CA, IL1B, FAS, CHUK, PIK3CG, ICAM1, IL6, MAP2K1, RELA, CREB1, FADD, MAPK1, JUN, MAPK14, IKBKG, MAPK9, MAPK8, IKBKB, SELE
HIF-1 signaling pathway	24	3.27E-16	PRKCA, PIK3CG, IL6, MAP2K1, RELA, EDN1, IGF1, NFKB1, TLR4, AKT1, IGF1R, MAPK1, CDKN1A, HIF1A, INS, BCL2, IFNG, SERPINE1, VEGFA, PIK3CA, NOS3, NOS2, INSR, NPPA
PI3K-Akt signaling pathway	39	1.97E-14	HSP90AB1, HRAS, PDGFB, TLR2, NFKB1, TLR4, PTEN, AKT1, IGF1R, INS, BCL2, ITGAV, PIK3CA, NOS3, INSR, FGF2, CHUK, PRKCA, PIK3CG, IL6, HSP90AA1, MAP2K1, CREB1, RELA, RXRA, TP53, IGF1, CDK6, CDK2, KDR, MAPK1, CDKN1A, GSK3B, VEGFA, IKBKG, COL1A1, JAK3, IKBKB, IL2
FoxO signaling pathway	25	5.16E-14	PIK3CG, IL6, HRAS, MAP2K1, TGFBR1, PRKAB1, IGF1, PTEN, TGFB1, CDK2, AKT1, CCNB1, IGF1R, MAPK1, PRMT1, CDKN1A, INS, MAPK14, MAPK9, PIK3CA, MAPK8, CAT, IKBKB, INSR, CHUK
Toll-like receptor signaling pathway	22	2.74E-13	PIK3CG, IL6, TNF, MAP2K1, RELA, TLR2, CXCL8, NFKB1, TLR4, FADD, STAT1, AKT1, MAPK1, JUN, MAPK14, IKBKG, MAPK9, IL1B, PIK3CA, MAPK8, IKBKB, CHUK
Osteoclast differentiation	24	2.81E-13	PIK3CG, TNF, MAP2K1, CREB1, TGFBR1, RELA, PPARG, NFKB1, STAT1, TGFB1, AKT1, TNFRSF1A, MAPK1, TNFSF11, JUN, MAPK14, IKBKG, IFNG, MAPK9, IL1B, PIK3CA, MAPK8, IKBKB, CHUK

Prolactin signaling pathway	18	2.19E-12	PIK3CG, HRAS, MAP2K1, RELA, NFKB1, ESR2, STAT1, SRC, AKT1, MAPK1, CYP17A1, TNFSF11, INS, GSK3B, MAPK14, MAPK9, PIK3CA, MAPK8
Apoptosis	17	2.87E-12	PIK3CG, TNF, AIFM1, RELA, TP53, NFKB1, FADD, AKT1, TNFRSF1A, CASP3, BAX, BCL2, IKBKG, PIK3CA, FAS, IKBKB, CHUK
NOD-like receptor signaling pathway	15	8.56E-11	HSP90AB1, IL6, TNF, HSP90AA1, RELA, CXCL8, NFKB1, MAPK1, MAPK14, IKBKG, IL1B, MAPK9, MAPK8, IKBKB, CHUK
Sphingolipid signaling pathway	20	2.43E-10	PRKCA, PIK3CG, HRAS, TNF, MAP2K1, RELA, TP53, NFKB1, PTEN, AKT1, TNFRSF1A, MAPK1, BCL2, BAX, MAPK14, MAPK9, PIK3CA, ABCC1, NOS3, MAPK8
Adipocytokine signaling pathway	15	2.68E-09	PPARA, TNF, RXRA, RELA, PRKAB1, NFKB1, ADIPOQ, LEP, AKT1, TNFRSF1A, IKBKG, MAPK9, MAPK8, IKBKB, CHUK
NF-κB signaling pathway	16	6.19E-09	ICAM1, TNF, PTGS2, RELA, CXCL8, TLR4, NFKB1, VCAM1, TNFRSF1A, TNFSF11, BCL2, IKBKG, IL1B, IKBKB, CHUK, PLAU
T cell receptor signaling pathway	17	9.12E-09	PIK3CG, HRAS, TNF, MAP2K1, RELA, NFKB1, AKT1, MAPK1, JUN, GSK3B, MAPK14, IFNG, IKBKG, PIK3CA, IKBKB, CHUK, IL2
VEGF signaling pathway	13	4.66E-08	PRKCA, PIK3CG, AKT1, MAPK1, HRAS, MAP2K1, PTGS2, MAPK14, VEGFA, PIK3CA, NOS3, SRC, KDR
MAPK signaling pathway	25	4.88E-08	PRKCA, HRAS, TNF, PDGFB, MAP2K1, TGFBR1, RELA, TP53, NFKB1, TGFB1, AKT1, TNFRSF1A, MAPK1, CASP3, JUN, MAPK14, IKBKG, MAPK9, IL1B, MAPK8, FAS, IKBKB, CACNA1C, FGF2, CHUK
Progesterone-mediated oocyte	15	5.07E-08	HSP90AB1, PIK3CG, HSP90AA1, MAP2K1, IGF1, CDK2, AKT1, CCNB1,

maturation			IGF1R, MAPK1, INS, MAPK14, MAPK9, PIK3CA, MAPK8
Neurotrophin signaling pathway	17	8.50E-08	PIK3CG, HRAS, MAP2K1, RELA, TP53, NFKB1, AKT1, MAPK1, JUN, BAX, MAPK14, GSK3B, BCL2, MAPK9, PIK3CA, MAPK8, IKBKB
Ras signaling pathway	23	1.02E-07	PRKCA, PIK3CG, HRAS, PDGFB, MAP2K1, RELA, IGF1, NFKB1, KDR, AKT1, IGF1R, MAPK1, INS, IKBKG, VEGFA, PLA2G2A, MAPK9, PIK3CA, MAPK8, IKBKB, FGF2, INSR, CHUK
B cell receptor signaling pathway	13	1.96E-07	PIK3CG, AKT1, MAPK1, HRAS, MAP2K1, RELA, JUN, GSK3B, IKBKG, PIK3CA, NFKB1, IKBKB, CHUK
ErbB signaling pathway	14	3.80E-07	PRKCA, PIK3CG, HRAS, MAP2K1, SRC, AKT1, MAPK1, CDKN1A, JUN, GSK3B, PIK3CA, MAPK9, MAPK8, AREG
Focal adhesion	21	4.23E-07	PRKCA, PIK3CG, HRAS, PDGFB, MAP2K1, IGF1, PTEN, SRC, KDR, AKT1, IGF1R, MAPK1, ITGAV, JUN, BCL2, GSK3B, VEGFA, MAPK9, PIK3CA, MAPK8, COL1A1
Fc epsilon RI signaling pathway	12	1.37E-06	PRKCA, PIK3CG, AKT1, MAPK1, HRAS, TNF, MAP2K1, MAPK14, PIK3CA, MAPK9, IL13, MAPK8
Estrogen signaling pathway	14	1.74E-06	HSP90AB1, PIK3CG, HRAS, HSP90AA1, MAP2K1, CREB1, MMP9, ESR2, SRC, AKT1, MAPK1, JUN, PIK3CA, NOS3
Rap1 signaling pathway	20	2.44E-06	PRKCA, PIK3CG, ITGAL, HRAS, PDGFB, MAP2K1, DRD2, IGF1, SRC, KDR, AKT1, IGF1R, MAPK1, INS, CNR1, MAPK14, VEGFA, PIK3CA, FGF2, INSR
Thyroid hormone signaling pathway	14	8.61E-06	PRKCA, PIK3CG, HRAS, MAP2K1, RXRA, TP53, STAT1, SRC, AKT1, MAPK1, HIF1A, ITGAV, GSK3B, PIK3CA

p53 signaling pathway	11	8.89E-06	CCNB1, CDKN1A, CASP3, BAX, SERPINE1, TP53, IGF1, CDK6, FAS, PTEN, CDK2
Bile secretion	11	1.16E-05	ABCB11, HMGCR, RXRA, SLCO1B1, ABCC4, ABCB1, ABCC2, CA2, NR1H4, ABCG2, SLC10A1
RIG-I-like receptor signaling pathway	11	1.33E-05	TNF, MAPK14, RELA, IKBKG, CXCL8, MAPK9, NFKB1, MAPK8, FADD, IKBKB, CHUK
cAMP signaling pathway	18	1.72E-05	PIK3CG, PPARA, ACOX1, MAP2K1, DRD2, CREB1, RELA, NFKB1, GLI1, AKT1, MAPK1, ADRB2, JUN, MAPK9, ABCC4, PIK3CA, MAPK8, CACNA1C
AMPK signaling pathway	14	1.81E-05	PIK3CG, HMGCR, CREB1, PPARG, FBP1, PRKAB1, IGF1, ADIPOQ, LEP, AKT1, IGF1R, INS, PIK3CA, INSR
mTOR signaling pathway	10	1.83E-05	PRKCA, PIK3CG, AKT1, MAPK1, TNF, INS, PIK3CA, IGF1, IKBKB, PTEN
Insulin signaling pathway	14	6.73E-05	PIK3CG, HRAS, MAP2K1, FBP1, PRKAB1, AKT1, MAPK1, INS, GSK3B, MAPK9, PIK3CA, MAPK8, IKBKB, INSR
Chemokine signaling pathway	16	1.13E-04	PIK3CG, HRAS, MAP2K1, RELA, CXCL8, NFKB1, STAT1, SRC, AKT1, MAPK1, GSK3B, IKBKG, PIK3CA, JAK3, IKBKB, CHUK
ABC transporters	8	1.36E-04	ABCB11, ABCC4, ABCC1, ABCB1, ABCC2, ABCA1, ABCG1, ABCG2
Cholinergic synapse	12	1.57E-04	PRKCA, PIK3CG, AKT1, MAPK1, ACHE, HRAS, CHRM5, MAP2K1, BCL2, CREB1, PIK3CA, CACNA1C
Inflammatory mediator regulation of TRP channels	11	2.47E-04	PRKCA, PIK3CG, TRPV1, MAPK14, TRPA1, PIK3CA, MAPK9, IGF1, IL1B, MAPK8, SRC
Natural killer cell mediated	12	3.62E-04	PRKCA, PIK3CG, ICAM1, MAPK1, ITGAL, CASP3, HRAS, TNF, MAP2K1,

cytotoxicity			IFNG, PIK3CA, FAS
Aldosterone-regulated sodium reabsorption	7	5.01E-04	PRKCA, PIK3CG, MAPK1, INS, PIK3CA, IGF1, INSR
Regulation of lipolysis in adipocytes	8	6.31E-04	PIK3CG, AKT1, ADRB2, PTGS2, INS, PTGS1, PIK3CA, INSR
GnRH signaling pathway	10	6.36E-04	PRKCA, MAPK1, HRAS, MAP2K1, MAPK14, JUN, MAPK9, MAPK8, CACNA1C, SRC
Serotonergic synapse	11	6.75E-04	PRKCA, MAPK1, APP, CASP3, HRAS, PTGS2, MAP2K1, MAOA, PTGS1, ALOX5, CACNA1C
Oxytocin signaling pathway	13	9.40E-04	PRKCA, PIK3CG, HRAS, PTGS2, MAP2K1, PRKAB1, SRC, MAPK1, CDKN1A, JUN, PIK3CA, NOS3, CACNA1C
Cytokine-cytokine receptor interaction	16	1.09E-03	IL6, TNF, PDGFB, TGFBR1, CXCL8, IL13, TGFB1, KDR, LEP, TNFRSF1A, TNFSF11, IFNG, VEGFA, IL1B, FAS, IL2
Signaling pathways regulating pluripotency of stem cells	12	1.16E-03	PIK3CG, AKT1, MAPK1, IGF1R, HRAS, MAP2K1, MAPK14, GSK3B, PIK3CA, IGF1, JAK3, FGF2
cGMP-PKG signaling pathway	13	1.44E-03	PIK3CG, AKT1, EDNRB, MAPK1, ADRB2, MAP2K1, INS, CREB1, PIK3CA, NOS3, CACNA1C, INSR, ADRA1D
PPAR signaling pathway	8	1.85E-03	PPARA, ACOX1, PPARD, RXRA, PPARG, ADIPOQ, MMP1, NR1H3
Steroid hormone biosynthesis	7	4.10E-03	CYP3A4, CYP17A1, CYP1B1, CYP1A1, CYP2E1, CYP1A2, CYP19A1
Retrograde endocannabinoid signaling	9	5.15E-03	PRKCA, MAPK1, PTGS2, MAPK14, CNR1, FAAH, MAPK9, MAPK8, CACNA1C
Adrenergic signaling in cardiomyocytes	11	5.23E-03	PRKCA, PIK3CG, AKT1, MAPK1, ADRB2, MAPK14, BCL2, CREB1, PIK3CA, CACNA1C, ADRA1D

Arachidonic acid metabolism	7	5.71E-03	CBR1, PTGIS, PTGS2, PTGS1, PLA2G2A, ALOX5, CYP2E1
Cytosolic DNA-sensing pathway	7	6.67E-03	IL6, RELA, IKBKG, IL1B, NFKB1, IKBKB, CHUK
Dopaminergic synapse	10	6.71E-03	PRKCA, AKT1, DRD2, MAPK14, CREB1, GSK3B, MAOA, MAPK9, MAPK8, CACNA1C
Gap junction	8	8.51E-03	PRKCA, MAPK1, HRAS, PDGFB, MAP2K1, DRD2, SRC, TUBB3
Arginine and proline metabolism	6	9.96E-03	LAP3, ODC1, NOS1, MAOA, NOS3, NOS2
Regulation of actin cytoskeleton	13	9.97 E-03	PIK3CG, ITGAL, MAPK1, HRAS, CHRM5, MAP2K1, PDGFB, INS, ITGAV, F2, PIK3CA, FGF2, SRC

Supplementary Table S7 Non-disease associated pathways of synthetic drugs used for treatment of ulcerative colitis (UC).

Term	Count	*P*-Value	Genes
Osteoclast differentiation	10	1.78E-07	TNF, NCF4, PLCG2, PPARG, IFNG, IL1B, NFKB2, IKBKB, IL1A, CHUK
TNF signaling pathway	9	4.50E-07	ICAM1, IL6, NOD2, TNF, MMP9, IL1B, IKBKB, CHUK, CXCL10
NF-κB signaling pathway	7	2.38E-05	ICAM1, TNF, PLCG2, IL1B, NFKB2, IKBKB, CHUK
NOD-like receptor signaling pathway	6	3.16E-05	IL6, NOD2, TNF, IL1B, IKBKB, CHUK
Antigen processing and presentation	6	1.51E-04	CIITA, TNF, RFX5, IFNG, RFXANK, RFXAP
Toll-like receptor signaling pathway	6	7.10E-04	IL6, TNF, IL1B, IKBKB, CHUK, CXCL10
Cytosolic DNA-sensing pathway	5	8.86E-04	IL6, IL1B, IKBKB, CHUK, CXCL10
Intestinal immune network for IgA production	4	3.88E-03	IL6, ITGB7, MADCAM1, ITGA4
HIF-1 signaling pathway	5	4.25E-03	IL6, PLCG2, IFNG, RPS6KB1, MTOR
Cytokine-cytokine receptor interaction	7	4.36E-03	IL6, TNF, IFNG, IL1B, IL13, IL1A, CXCL10
mTOR signaling pathway	4	7.01E-03	TNF, RPS6KB1, MTOR, IKBKB
Leukocyte transendothelial migration	5	8.19E-03	ICAM1, MMP9, NCF4, PLCG2, ITGA4
